# Fully‑automated deep‑learning segmentation of pediatric cardiovascular magnetic resonance of patients with complex congenital heart diseases

**DOI:** 10.1186/s12968-020-00678-0

**Published:** 2020-11-30

**Authors:** Saeed Karimi-Bidhendi, Arghavan Arafati, Andrew L. Cheng, Yilei Wu, Arash Kheradvar, Hamid Jafarkhani

**Affiliations:** 1 Center for Pervasive Communications and Computing, University of California, Irvine, Irvine, USA; 2 Edwards Lifesciences Center for Advanced Cardiovascular Technology, University of California, Irvine, Irvine, USA; 3 The Keck School of Medicine, University of Southern California and Children’s Hospital Los Angeles, Los Angeles, USA

**Keywords:** Complex CHD analysis, CMR image analysis, Fully convolutional networks, Generative adversarial networks, Deep learning, Machine learning

## Abstract

**Background:**

For the growing patient population with congenital heart disease (CHD), improving clinical workflow, accuracy of diagnosis, and efficiency of analyses are considered unmet clinical needs. Cardiovascular magnetic resonance (CMR) imaging offers non-invasive and non-ionizing assessment of CHD patients. However, although CMR data facilitates reliable analysis of cardiac function and anatomy, clinical workflow mostly relies on manual analysis of CMR images, which is time consuming. Thus, an automated and accurate segmentation platform exclusively dedicated to pediatric CMR images can significantly improve the clinical workflow, as the present work aims to establish.

**Methods:**

Training artificial intelligence (AI) algorithms for CMR analysis requires large annotated datasets, which are not readily available for pediatric subjects and particularly in CHD patients. To mitigate this issue, we devised a novel method that uses a generative adversarial network (GAN) to synthetically augment the training dataset via generating synthetic CMR images and their corresponding chamber segmentations. In addition, we trained and validated a deep fully convolutional network (FCN) on a dataset, consisting of $64$ pediatric subjects with complex CHD, which we made publicly available. Dice metric, Jaccard index and Hausdorff distance as well as clinically-relevant volumetric indices are reported to assess and compare our platform with other algorithms including U-Net and cvi42, which is used in clinics.

**Results:**

For congenital CMR dataset, our FCN model yields an average Dice metric of $91.0\mathrm{\%}$ and $86.8\mathrm{\%}$ for LV at end-diastole and end-systole, respectively, and $84.7\mathrm{\%}$ and $80.6\mathrm{\%}$ for RV at end-diastole and end-systole, respectively. Using the same dataset, the cvi42, resulted in $73.2\mathrm{\%}$, $71.0\mathrm{\%}$, $54.3\mathrm{\%}$ and $53.7\mathrm{\%}$ for LV and RV at end-diastole and end-systole, and the U-Net architecture resulted in $87.4\mathrm{\%}$, $83.9\mathrm{\%}$, $81.8\mathrm{\%}$ and $74.8\mathrm{\%}$ for LV and RV at end-diastole and end-systole, respectively.

**Conclusions:**

The chambers’ segmentation results from our fully-automated method showed strong agreement with manual segmentation and no significant statistical difference was found by two independent statistical analyses. Whereas cvi42 and U-Net segmentation results failed to pass the t-test. Relying on these outcomes, it can be inferred that by taking advantage of GANs, our method is clinically relevant and can be used for pediatric and congenital CMR segmentation and analysis.

## Background

Congenital heart diseases (CHDs) are the most common among the birth defects [1]. It is currently estimated that $83\mathrm{\%}$ of newborns with CHD in the U.S. survive infancy [2]. These patients require routine imaging follow ups. Cardiovascular magnetic resonance (CMR) imaging is the imaging modality of choice for assessment of cardiac function and anatomy in children with CHD. Not only does CMR deliver images with high spatial and acceptable temporal resolution, but also it is non-invasive and non-ionizing [3, 4]. On the other hand, CMR analysis in pediatric CHD patients is among the most challenging, time-consuming, and operator-intensive clinical tasks.

Presently, artificial intelligence (AI) and particularly deep-learning show strong promise for automatic segmentation of CMR images [5–8]. While the current AI-based methods have been successfully used for delineating the adult heart disease, they are not yet reliable for segmenting the CMR images of CHD patients, and particularly in children [8, 9]. The foremost basis for this shortcoming is the anatomical heterogeneity and lack of large CMR databases that include data from a diverse group of CHD subjects acquired by diverse scanners and pulse sequences. As indicated by Bai et al. [7], a major limitation of the existing learning methods is the use of homogeneous datasets where the majority of the CMR data are from adult subjects with healthy or closely mimicking healthy hearts, e.g., the Second Annual Data Science Bowl [10] and UK CMR Biobank [11], among others [12, 13].

Training neural networks requires a large set of data that does not currently exist for complex CHD subjects. Another limitation is overfitting, especially over training, to image patterns in a specific dataset that includes images from the same scanner model/vendor, as also reported by Bai et al. [7]. Dealing with limited data is a major challenge in designing effective neural networks for pediatric CMR, particularly for CHD subjects, and necessitates innovative approaches [9].

Among the learning-based algorithms, supervised deep-learning is currently considered the state-of-the-art for CMR segmentation [14]. Nevertheless, major limitations of deep-learning methods are their dependency on the number of manually-annotated training data [15]. Small datasets can incur a large bias, which makes these methods ineffective and unreliable when the heart shape is outside the learning set, as frequently observed in CHD subjects.

To mitigate the need for large datasets of manually-annotated CHD data, in this study, we employ a Deep Convolutional Generative Adversarial Network (DCGAN) [16] that generates synthetically segmented CMR images and further enriches the training data beyond the classical affine transformations. DCGAN has enabled our deep-learning algorithms to successfully and accurately segment CMR images of complex CHD subjects beyond the existing AI methods.

## Methods

### Dataset

Our dataset includes $64$ CMR studies from pediatric patients with an age range of $2$ to $18$ scanned at the Children’s Hospital Los Angeles (CHLA). The CMR dataset includes scans from patients with Tetralogy of Fallot (TOF; $\mathrm{n }= 20$), Double Outlet Right Ventricle (DORV; $\mathrm{n }= 9$), Transposition of the Great Arteries (TGA; $\mathrm{n }= 9$), Cardiomyopathy ($\mathrm{n }= 8$), Coronary Artery Anomaly (CAA; $\mathrm{n }= 9$), Pulmonary Stenosis or Atresia ($\mathrm{n }= 4$), Truncus Arteriosus ($\mathrm{n }= 3$), and Aortic Arch Anomaly ($\mathrm{n }= 2$). All TGA cases were D-type but had been repaired with an arterial switch operation. The study was reviewed by the Children’s Hospital Los Angeles Institutional Review Board and was granted an exemption per 45 CFR 46.104[d] [4][iii] and a waiver of HIPAA authorization per the Privacy Rule (45 CFR Part 160 and Subparts A and E of Part 164).

### CMR studies

Imaging studies were performed on either a 1.5 T (Achieva, Philips Healthcare, Best, the Netherlands) or at 3 T (Ingenia, Philips Healthcare). CMR images for ventricular volume and function analysis were obtained using a standard balanced steady state free precession (bSSFP) sequence without any contrast. Each dataset includes $12 - 15$ short-axis slices encompassing both right ventricle (RV) and left ventricle (LV) from base to apex with $20 - 30$ frames per cardiac cycle. Typical scan parameters were slice thickness of $6 - 10\mathrm{mm}$, in-plane spatial resolution of $1.5 - 2{\mathrm{mm}}^{2}$, repetition time of $3 - 4\mathrm{ms}$, echo time of $1.5 - 2\mathrm{ms}$, and flip angle of $60$ degrees. Images were obtained with the patients free breathing; $3$ signal averages were obtained to compensate for respiratory motion. Manual image segmentation was performed by a board-certified pediatric cardiologist sub-specialized in CMR with experience consistent with Society for Cardiovascular Magnetic Resonance (SCMR) Level 3 certification. Endocardial contours were drawn on end-diastolic and end-systolic images. Ventricular volumes and ejection fraction were then computed from these contours. Manual annotations were performed according to SCMR guidelines with cvi42 software (Circle Cardiovascular Imaging, Calgary, Alberta, Canada) without the use of automated segmentation tools. The ventricular cavity in the basal slice was identified by evaluating wall thickening and cavity shrinking in systole.

### Post-processing of CMR data

The original image size was $512\times 512$ pixels. The original dataset was first preprocessed by center-cropping each image to the size of $445\times 445$ to remove patients’ identifiers. Subsequently, all images were examined to ensure that both the heart and segmentation mask are present. To reduce the dimensionality, each cropped image was subsequently resized to $128\times 128$ using the *imresize* function in the open-source Python library SciPy. The entire process was performed using two different down-sampling methods: (1) nearest-neighbor down-sampling and (2) bi-cubical down-sampling. For training data, twenty-six patients ($10$ TOFs, $4$ DORVs, $4$ TGAs, $4$ CAAs and $4$ cardiomyopathy patients) were randomly selected whereas the remaining $38$ patients were used as test data.

### Image segmentation using fully convolutional networks

A fully convolutional network (FCN), in comparison with a U-net [17] and cvi42, was used for automated pixelwise image segmentation. Convolutional networks are a family of artificial neural networks that are comprised of a series of convolutional and pooling layers in which the data features are learned in various levels of abstraction. These networks are mostly useful when the data is either an image or a map such that the proximity among pixels represents how associated they are. Examples of FCNs used for segmenting healthy adult CMR images include [7, 18]. While these FCNs yield good segmentation accuracy for healthy adult CMR images, they show poor performance on CHD subjects [7]. Inspired by the “skip” architecture used by Long et al. [19] and the FCN model introduced by Tran [18], we designed a novel $19-$ layer FCN for an automated pixelwise image segmentation in CHD subjects.

#### FCN architecture

**Fig. 1:**
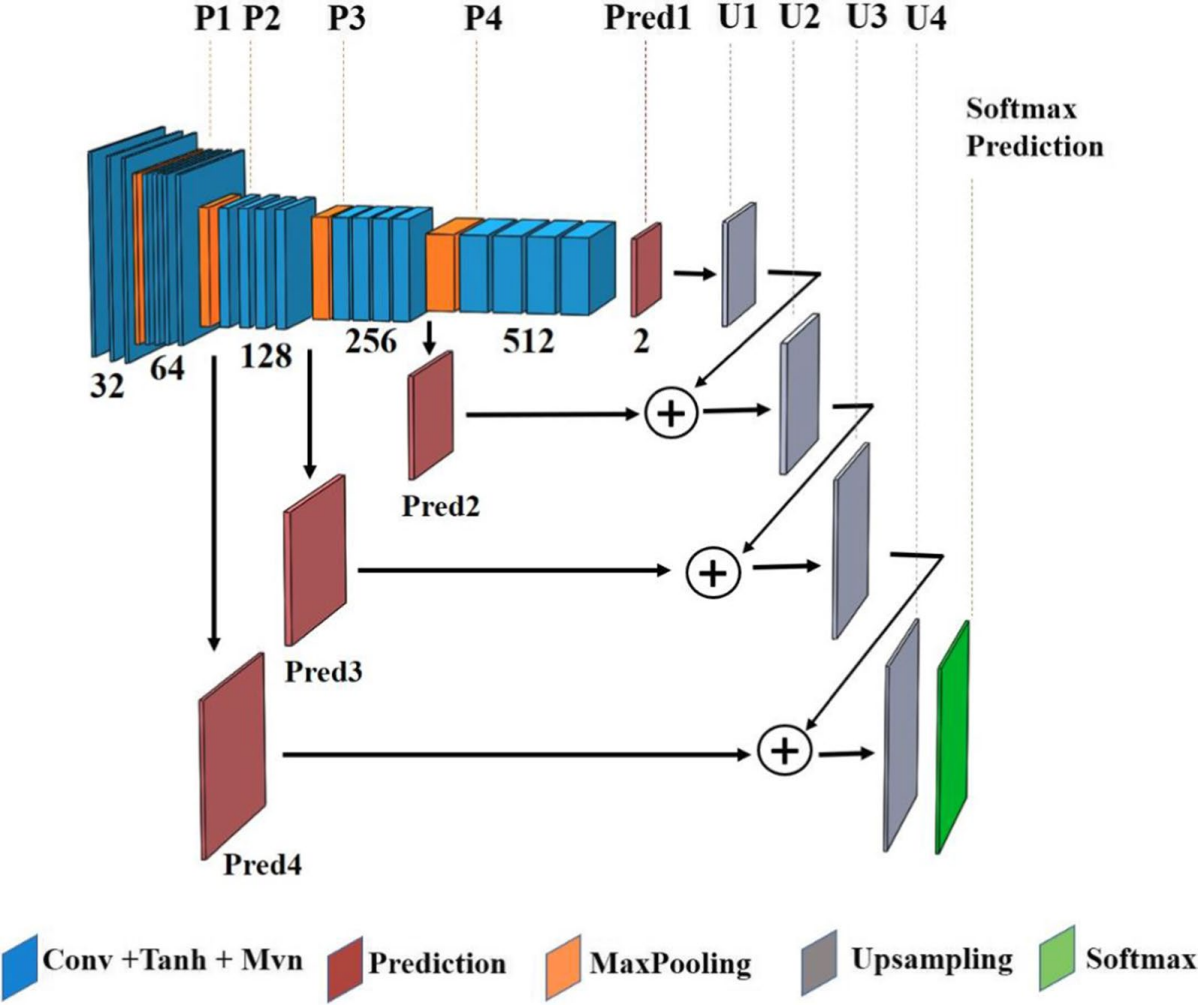
Fully convolutional network (FCN) architecture

The design architecture of our $19-$ layer FCN model and the number of filters for each convolution layer are specified in Fig. 1; four max-pooling layers with pooling size of $3$ are employed to reduce the dimension of the previous layer’s output. Fine and elementary visual features of an image, e.g., the edges and corners, are learned in the network’s shallow layers whereas the coarse semantic information is generated over the deeper layers. These coarse and fine features are combined to learn the filters of the up-sampling layers, which are transposed convolution layers with the kernel size of $4$. The FCN’s input is a $128\times 128$ image and the network’s output is a $128\times 128$ dense heatmap, predicting class membership for each pixel of the input image. The technical details of the FCN architecture are fully described in the Appendix.

Despite incorporating ${l}_{2}-$ regularization and dropout in the FCN architecture, as explained in the Appendix, overfitting was still present due to the lack of a large set of annotated training data. A standard solution to this problem is to artificially augment the training dataset using various known image transformations [20]. Classic data augmentation techniques include affine transformations such as rotation, flipping, and shearing [21]. To conserve the characteristics of the heart chambers, only rotation and flipping were used and the transformations such as shearing that instigate shape deformation were avoided. Each image was first rotated $10$ times at the angles $\theta =\left[{0}^{^\circ }, {20}^{^\circ },{40}^{^\circ }, ..., {180}^{^\circ }\right]$. Subsequently, each rotated image either remained the same or flipped horizontally, vertically, or both. As a result of this augmentation, the number of training data was multiplied by a factor of $10\times 4=40$.

#### FCN training procedure

The dataset was randomly split into training/validation with the ratio of $0.8/0.2$. The validation set was used to provide an unbiased performance estimate of the final tuned model when evaluated over unseen data. Each image was then normalized to zero-mean and unit-variance. Network parameters were initialized according to the Glorot’s uniform scheme [22].

To learn the model parameters, stochastic gradient descent (SGD) with learning rate of $0.002$ and moment of $0.9$ was used to accelerate SGD in the relevant direction and dampen oscillations. To improve the optimization process, Nesterov moment updates [23] were used for assessing the gradient at the “look-ahead” position instead of the current position. The network was trained using a batch size of $5$ for $450$ epochs, i.e., passes over the training dataset, to minimize the negative dice coefficient between the predicted and manual ground-truth segmentation.

### Deep convolutional generative adversarial networks to synthesize CMR images

While classic data augmentation techniques increased the number of training data by a factor of $40$, it did not solve the overfitting issue. To mitigate that, generative adversarial networks (GANs) were used to artificially synthesize CMR images and their corresponding chambers’ segmentation. GANs are a specific family of generative models used to learn a mapping from a known distribution, e.g., random noise, to the data distribution.

A DCGAN was designed to synthesize CMR images to augment the training data. The architecture of both generator and discriminator networks along with their training procedures are described next.

#### DCGAN architecture

**Fig. 2:**
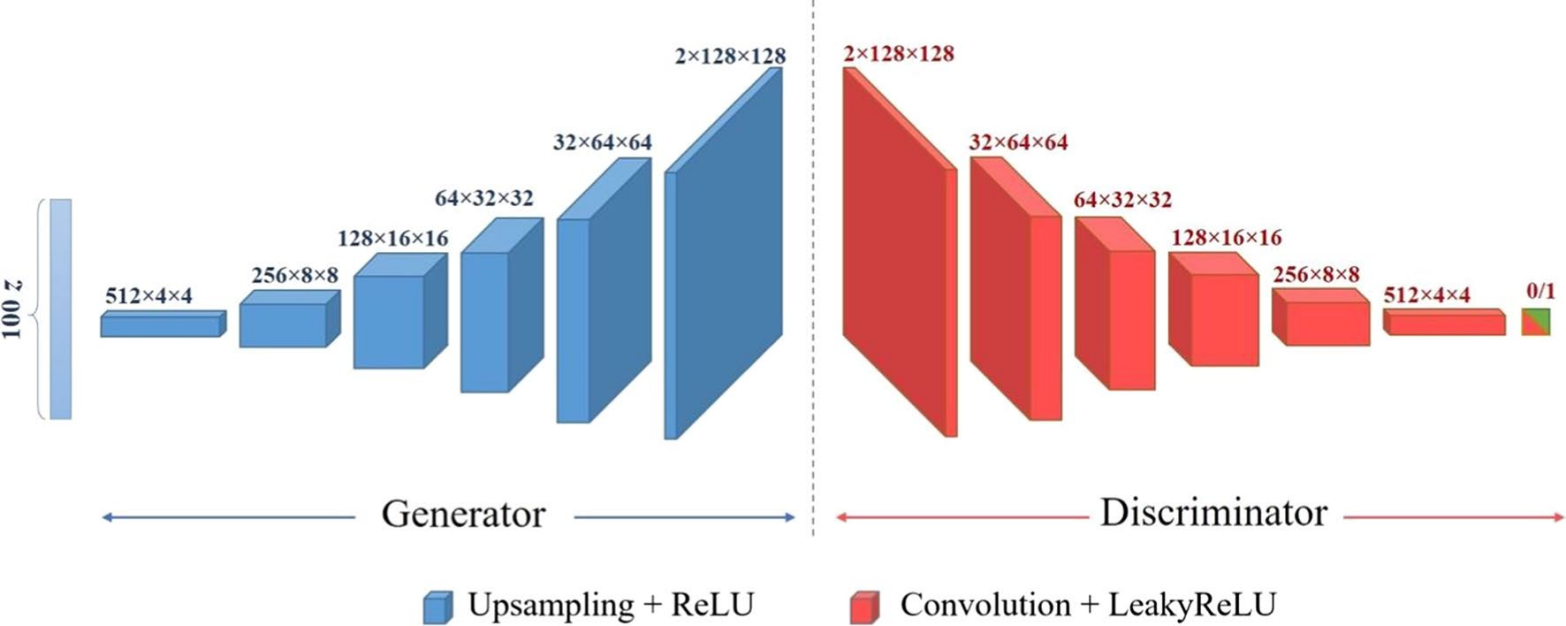
Deep convolutional generative adversarial network (DCGAN) architecture

The generator’s architecture is shown in Fig. 2. The input to the generator network is a random noise $z\in {\mathbb{R}}^{100}$ drawn from a standard normal distribution $\mathcal{N}\left(0,\mathbf{I}\right)$. The input is passed through six 2D transposed convolution, also known as fractionally-strided convolution, layers with kernel size of $4\times 4$ to up-sample the input into a $128\times 128$ image. In the first transposed convolution layer, a stride of $1$ pixel is used while a stride of $2$ pixels is applied to the cross-correlation in the remaining layers. The number of channels for each layer is shown in Fig. 2. All 2D transposed convolution layers except the last one are followed by a rectified linear unit (ReLU) layer. The last layer is accompanied by a Tanh activation function. The generator network’s output includes two channels where the first is used for the synthetic CMR image and the second contains the corresponding chamber’s segmentation mask.

The discriminator network’s architecture is a deep convolutional neural network (CNN) as shown in Fig. 2. The discriminator network’s input is a $2\times 128\times 128$ image whose output is a scalar representing the probability that the input is a real pair of image with its corresponding segmentation mask. The model includes six 2D convolution layers with kernel size of $4\times 4$ and stride of $2$ pixels except for the last layer for which a $1-$ pixel stride value is used. The number of channels for each convolution layer is shown in Fig. 2. All layers except the last one are followed by a Leaky ReLU layer with negative slope value of $0.2$. The last layer is accompanied by a sigmoid function.

#### DCGAN training procedure

The training data was normalized to zero-mean and unit-variance to stabilize the DCGAN learning process. Each training sample was then rotated $19$ times at angles $\theta =\left[{0}^{^\circ },{10}^{^\circ },{20}^{^\circ },...,{180}^{^\circ }\right]$ while each rotated image either remained the same or flipped horizontally, vertically or both. As a result of this augmentation process, the number of training data was multiplied by a factor of $19\times 4=76$.

The DCGAN’s two known issues are mode collapse and gradient vanishing [24]. Mode collapse attributes to the case in which too many values of the input noise are mapped to the same value in the data space. This happens when the generator is over-trained with respect to the discriminator. Alternatively, gradient vanishing refers to the situation in which the discriminator becomes too successful in distinguishing the real from synthetic images with no gradient is backpropagated to the generator. In this case, the generator network cannot learn to generate synthetic images that are similar to the real images. To address these concerns, first, the network parameters were initialized according to a Gaussian distribution with zero-mean and variance of $0.02$. To learn the network parameters, Adam optimizer [25] was used for both generator and discriminator networks. Additional information is provided in the Appendix. Each iteration of the learning procedure included the following two steps:

First, a single optimization step was performed to update the discriminator: A batch of $5$ real image samples and their corresponding segmentation masks from the training data was randomly selected. Label $1$ was assigned to them since they are real samples. These pairs of real images and their masks were then passed through the discriminator network and the gradient of the loss, i.e., the binary cross entropy between predicted and true labels, was backpropagated to accordingly adjust the discriminator weights. Then, a batch of five noise samples was drawn from the standard normal distribution and passed through the generator network to create five pairs of images and their corresponding masks. These pairs were then labeled with $0$ since they were synthetic samples. This batch of synthetic data was then passed through the discriminator and the gradient of the loss was backpropagated to fine-tune the discriminator weights.

Second, an additional optimization step was performed to update the generator: Each pair of synthetic image and its corresponding segmentation mask from the previous step was labeled $1$ to mislead the discriminator and create the perception that the pair is real. These samples were then passed through the discriminator and the gradient of the loss was backpropagated to adjust the generator weights.

In summary, in the first step, the discriminator was fine-tuned while the generator was unchanged, and in the second step, the generator was trained while the discriminator remained unchanged. The training process continued for $40,000$ iterations, or until the model converged and an equilibrium between the generator and discriminator networks was established.

#### DCGAN post-processing

The pixel value in each real mask is either $1$ or $0$ implying whether each pixel belongs to one of the ventricles or not. Therefore, the value of each pixel in a synthesized chamber mask was quantized to $0$ when it was less than $0.5$ and rounded up to $1$ otherwise. To avoid very small or large mask areas, only the synthetic samples for which the ratio of the mask area to the total area was within a certain range were retained. For nearest-neighbor down-sampling, the range was between $0.005$ and $0.025$ while for the bi-cubical down-sampling, the range was between $0.02$ and $0.05$. Finally, the connected components in each binary mask were located using the MATLAB (Mathworks, Natick, Massachusetts, USA) function *bwconncomp*. If there were more than one connected component and the ratio of the area of the largest component to the second largest component was less than $20$, that pair of image and mask would be removed from the set of synthetically-generated data.

### Network training and testing

#### Fully convolutional networks using real dataset

For each chamber, one FCN was trained on the CMR images of $26$ patients and their augmentation via geometric transformations. Each model was jointly trained on both end-diastolic (ED) and end-systolic (ES) images for each heart chamber. These networks are called LV-FCN and RV-FCN in the results section.

#### Fully convolutional networks using synthetically augmented dataset

Two separate DCGAN models were designed for LV and RV to further augment the training data. The designed DCGAN was used to generate $6,000$ pairs of synthetic images and their corresponding segmentation masks. Applying the DCGAN post-processing step, a set of $2,500$ synthetic images, out of the $6,000$ generated pairs, was used for each chamber. Each of the $2,500$ selected images was then either remained the same, or flipped horizontally, vertically, or rotated $4$ times at angles $\theta =\left[{45}^{^\circ },{90}^{^\circ },{135}^{^\circ },{180}^{^\circ }\right]$. Thus, $\mathrm{2,500}\times 7=\mathrm{17,500}$ synthetic CMR images and their corresponding segmentation masks were generated for each ventricle. Finally, our synthetically augmented repertoire included the CMR images of $26$ patients and their augmentation via geometric transformations plus the generated $17,500$ synthetic CMR images. Using this synthetically augmented dataset, another FCN was trained for each chamber. Each model was jointly trained on both ED and ES images. The networks designed using the synthetically augmented dataset (SAD) are called LV-FCN-SAD and RV-FCN-SAD in the results section.

#### U-Net architecture

**Fig. 3:**
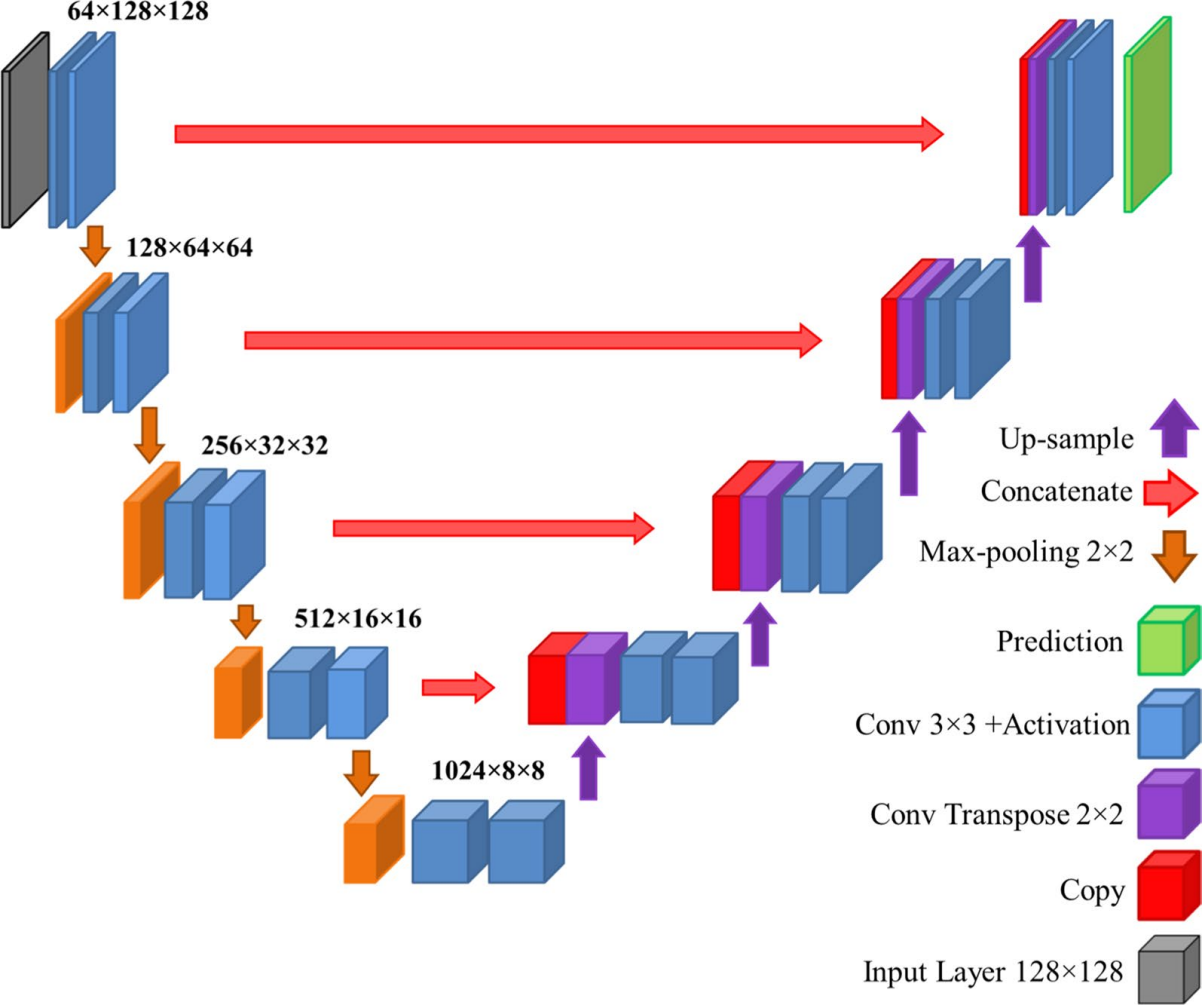
U-Net architecture

In addition to our network architecture described above, a traditional U-Net model was designed to compare its results with those of our designed FCN. For this purpose, a customized U-Net architecture with the input size of $128\times 128$ was used. The architecture of the U-Net model is shown in Fig. 3 and its code is available at https://github.com/karolzak/keras-unet. Similar to the case of our FCN, for each chamber, a network was trained on the training set of $26$ patients and its augmentation via geometric transformations. In the results section, these networks are referred to as LV-UNet and RV-UNet. For each chamber, another network was trained on the synthetically segmented CMR images, as was used for designing FCN-SAD. These networks are referred to as LV-UNet-SAD and RV-UNet-SAD. Each network was jointly trained on both ED and ES images for each chamber.

#### Commercially available segmentation software

The results generated by our models were compared with the results from cvi42 (Circle Cardiovascular Imaging Inc) on our test set that included CMR images from $38$ patients. All volumetric measures were calculated using OsiriX Lite software (Pixmeo, Bernex, Switzerland). To calculate the volume, small modifications were applied to the open source plugin available at https://github.com/chrischute/numpy2roi to make the format consistent with our dataset. The segmented CMR images were converted into OsiriX’s .roi files using the modified plugin. The resulted .roi files were imported to the OsiriX Lite software for volume calculation through its built-in 3D construction algorithm.

Our method was developed using the Python 2.7.12 and performed on a workstation with Intel(R) Core (TM) i7 − 5930 K CPU 3.50 GHz with four NVIDIA GeForce GTX 980 Ti GPUs, on a 64 − bit Ubuntu platform.

#### Metrics for performance verification

Our results were compared head-to-head with U-Net and cvi42. Two different classes of metrics are used to compare the performance of cardiac chamber segmentation methods.

One class uses the clinical indices, such as volumetric data that are crucial for clinical decision making. These indices may not identify the geometric point-by-point differences between automated and manually delineated segmentations.

Another class of indices uses geometric metrics that indicate how mathematically close the automatic segmentation is to that of the ground-truth. These include the average Dice metric, Jaccard index, Hausdorff distance (HD) and mean contour distance (MCD).

#### Generalizability to additional training and test subjects

To evaluate the generalizability of our framework on subjects not included in our dataset, our method was tested on the 2017 MICCAI’s Automated Cardiac Diagnosis Challenge (ACDC). The ACDC dataset includes $100$ subjects: (i) healthy ($n = 20$); (ii) previous myocardial infarction ($n = 20$); (iii) dilated cardiomyopathy ($n = 20$); (iv) hypertrophic cardiomyopathy ($n = 20$); and (v) abnormal RV ($n = 20$). For a consistent image size, five subjects were removed and the remaining $95$ subjects were zero-padded to $256\times 256$, and then down-sampled to $128\times 128$ using nearest-neighbor down-sampling method. Three subjects from each group were randomly selected as training data and the remaining $80$ subjects were left as the test data.

For each chamber, one FCN was trained on the combined CMR images of both training sets, i.e. $26$ patients from our dataset and $15$ from the ACDC dataset, and their augmentation via geometric transformations. For each heart chamber, another FCN is trained on the dataset that is further augmented via previously generated set of synthetically segmented CMR images. Each model was jointly trained on both ED and ES images for each heart chamber. The first and second segmentation networks are referred to as FCN-2.0 and FCN-SAD-2.0, respectively. FCN-2.0 and FCN-SAD-2.0 were evaluated on the combined set of test subjects, i.e. $38$ patients from our dataset and $80$ patients from the ACDC dataset.

#### Statistical methods

Paired student t-test and intraclass correlation coefficient (ICC) were used for statistical analysis of predicted volumes. The p-value for the paired student t-test can be interpreted as the evidence against the null hypothesis that predicted and ground-truth volumes have the same mean values. A p-value greater than $0.05$ is considered as passing the statistical hypothesis testing. The intraclass correlation coefficient describes how strongly the measurements within the same group are similar to each other. The intraclass correlation first proposed by Fisher et al. [26] was used. It focuses on the paired predicted and ground-truth measurements. The guidelines proposed by Koo and Li [27] were used to interpret the ICC values, as defined below: (a) less than $0.5$: poor; (b) between $0.50$ and $0.75$: moderate; (c) between $0.75$ and $0.90$: good; and (d) more than $0.90$: excellent.

## Results

Characteristics of the cohort are reported first. Then, our synthetically generated CMR images and the corresponding automatically generated segmentation masks are presented. Different performance metrics and clinical indices for our fully automatic method compared to those of manual segmentation (ground-truth) are reported. In addition, the same indices calculated by cvi42 software and U-Net are presented for head-to-head performance comparison.

### Characteristics of the Cohort

Characteristics of the cohort are reported in Tables 1 and 2. All chamber volumes in these tables are calculated based on the manual delineation.

**Table 1:** Characteristics of the cohort (Volumes)

	n	Min	Max	Mean	Median
LVEDV (mL)					
Aortic Arch Anomaly	2	87.39	196.09	141.74	141.74
Cardiomyopathy	8	82.55	179.02	114.37	93.66
Coronary Artery Disease	9	21.43	123.24	79.72	89.55
DORV	9	10.23	126.6	44.8	40.05
Pulmonary Stenosis/Atresia	4	88.74	130.22	101.83	94.18
TGA	9	39.18	167.03	113.35	133.77
TOF	20	18.32	153.68	87.55	92.19
Truncus arteriosus	3	70.23	201.44	124.13	100.73
All	64	10.23	201.44	91.72	89.41
LVESV (mL)					
Aortic Arch Anomaly	2	22.06	68.3	45.18	45.18
Cardiomyopathy	8	15.07	80.2	41.1	32.5
Coronary Artery Disease	9	8.28	58.6	28.99	29.76
DORV	9	4.18	43.33	17.4	17.7
Pulmonary Stenosis/Atresia	4	31.56	53.28	38.25	34.09
TGA	9	15.94	68.86	45.62	46.71
TOF	20	5.95	69.01	34.21	33.22
Truncus arteriosus	3	27.88	90.48	55.28	47.47
All	64	4.18	90.48	35.16	31.66
RVEDV (mL)					
Aortic Arch Anomaly	2	100.34	215.08	157.71	157.71
Cardiomyopathy	8	78.94	180.94	121.31	114.3
Coronary Artery Disease	9	20.13	171.28	92.35	106.01
DORV	9	25.31	236.22	80.0	69.72
Pulmonary Stenosis/Atresia	4	126.2	264.54	176.92	158.48
TGA	9	42.58	179.98	121.33	138.93
TOF	20	28.63	265.7	137.12	129.67
Truncus arteriosus	3	99.15	201.42	147.0	140.43
All	64	20.13	265.7	122.19	115.43
RVESV (mL)					
Aortic Arch Anomaly	2	38.43	101.04	69.73	69.73
Cardiomyopathy	8	13.27	86.81	45.25	36.43
Coronary Artery Disease	9	8.49	70.26	34.04	33.57
DORV	9	6.37	112.31	35.91	34.51
Pulmonary Stenosis/Atresia	4	49.04	129.65	80.72	72.09
TGA	9	15.93	84.68	50.08	41.52
TOF	20	13.56	136.99	63.74	59.21
Truncus arteriosus	3	43.3	73.47	56.37	52.34
All	64	6.37	136.99	52.32	46.07

*DORV* double outlet right ventricle, *TGA* transposition of the great arteries; *TOF* tetralogy of Fallot

**Table 2:** Clinical characteristics of the cohort (Age, Weight, Height)

	n	Min	Max	Mean	Median
Age (years)					
Aortic Arch Anomaly	2	17.9	18.3	18.1	18.1
Cardiomyopathy	8	9.4	17.1	13.6	13.9
Coronary Artery Disease	9	1.1	19.8	9.8	11.5
DORV	9	0.5	13	6.9	7.5
Pulmonary Stenosis/Atresia	4	8.6	16.5	12.9	13.2
TGA	9	2.7	18.9	11.2	11.7
TOF	20	0.4	20.2	10.9	11.9
Truncus arteriosus	3	10.3	23.3	15	11.3
All	64	0.4	23.3	11.1	12
Weight (kg)					
Aortic Arch Anomaly	2	49.0	62.6	55.8	55.8
Cardiomyopathy	8	43.8	114.5	71.3	62.6
Coronary Artery Disease	9	12	79.3	36.9	43.3
DORV	9	7.1	63.0	23.3	23.0
Pulmonary Stenosis/Atresia	4	35.5	54.5	47.1	49.1
TGA	9	13	63	41.3	49.1
TOF	20	3.5	124.3	42.8	38.4
Truncus arteriosus	3	25.0	70.5	41.5	29.0
All	64	3.5	124.3	43.2	43.6
Height (cm)					
Aortic Arch Anomaly	2	142.0	179.0	160.5	160.5
Cardiomyopathy	8	137.0	181.0	160.0	160.0
Coronary Artery Disease	9	97.0	169.4	144.5	155.0
DORV	9	64.5	153.0	109.4	121.0
Pulmonary Stenosis/Atresia	4	136.0	162.0	152.3	155.5
TGA	9	88.0	172.0	138.1	148.0
TOF	20	57.5	174.0	133.8	142.0
Truncus arteriosus	3	133.0	173.0	153.0	153.0
All	64	57.5	181.0	138.4	145.0

### Real and synthetically generated CMR images

**Fig. 4:**
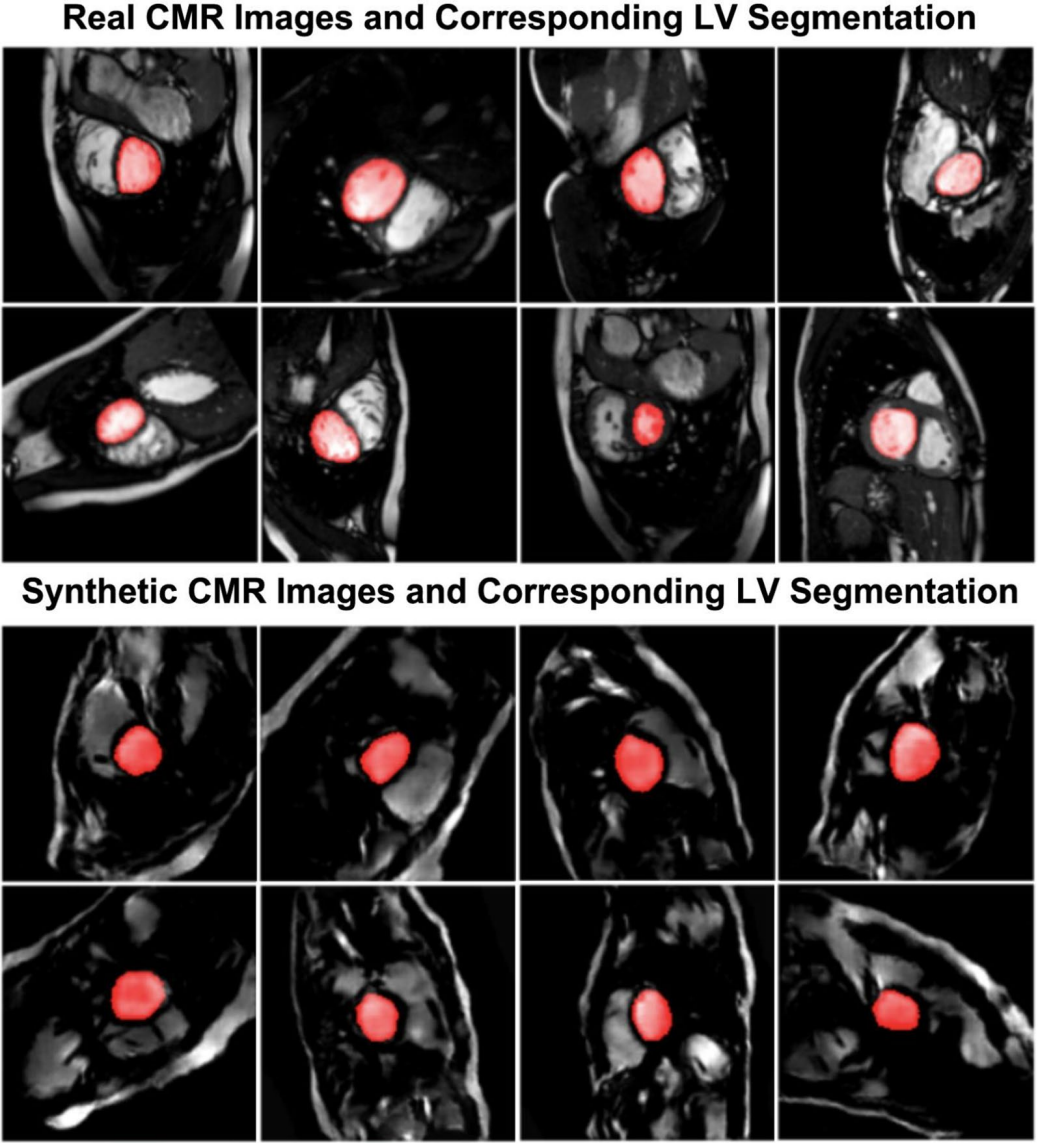
Sample segmented images for left ventricle (LV)

**Fig. 5:**
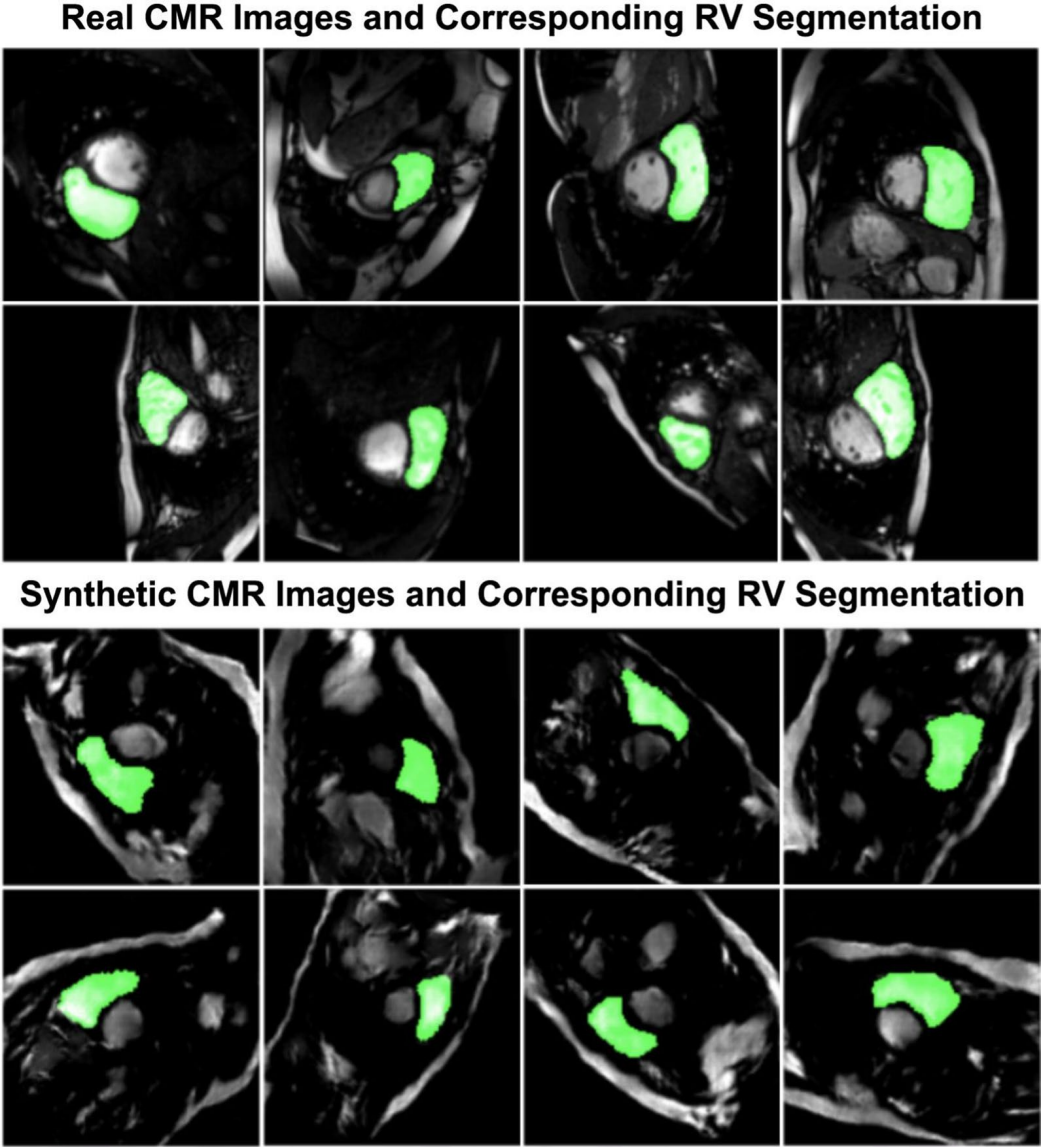
Sample segmented images for right ventricle (RV)

A sample batch of real CMR images, including their manually segmented LV masks is compared with a sample batch of synthetically generated CMR images with their corresponding automatically-generated LV masks in Fig. 4. Similar comparison is made for RV in Fig. 5.

### Segmentation performance

As mentioned in the method section, two separate down-sampling methods–nearest-neighbor and bi-cubical–were practiced and their training/testing were independently performed. The results for both methods are reported here:

#### Segmentation performance for nearest-neighbor down-sampling

The average Dice metric, Jaccard index, Hausdorff distance (HD), mean contour distance (MCD) and coefficient of determination ${R}_{vol}^{2}$ for FCN and FCN-SAD computed based on the ground-truth are reported in Table 3.

**Table 3:** Mean (SD) of different quantitative metrics for nearest-neighbor down-sampling

	Dice (%)	Jaccard (%)	HD (mm)	MCD (mm)	${\text{R}}_{{{\text{vol}}}}^{2} (\%)$
LVED					
FCN	86.5 (22.2)	80.7 (23.5)	6.9 (12.1)	2.3 (6.4)	98.5
FCN-SAD	**90.6 (13.8)**	**84.9 (16.5)**	**5.0 (7.4)**	**1.7 (3.8)**	**99.3**
cvi42	73.2 (34.3)	66.5 (33.0)	7.5 (13.6)	3.4 (10.8)	78.6
U-Net	84.5 (24.4)	78.4 (25.4)	7.2 (10.2)	3.3 (8.5)	93.4
U-Net-SAD	87.1 (21.9)	81.4 (22.6)	6.7 (10.8)	2.3 (7.1)	97.9
LVES					
FCN	83.2 (20.9)	75.1 (22.5)	6.9 (12.0)	2.7 (7.4)	93.0
FCN-SAD	**85.0 (18.8)**	**77.3 (21.2)**	**6.3 (9.4)**	**2.5 (5.6)**	**96.6**
cvi42	71.0 (32.2)	62.6 (30.5)	7.9 (15.3)	4.0 (13.3)	76.6
U-Net	79.4 (25.2)	71.2 (26.1)	7.1 (10.1)	2.7 (6.8)	82.2
U-Net-SAD	82.3 (20.9)	73.8 (22.5)	7.6 (11.9)	2.5 (6.7)	92.3
RVED					
FCN	80.3 (24.0)	71.9 (24.9)	14.2 (15.7)	6.6 (13.6)	87.0
FCN-SAD	**84.4 (20.2)**	**76.7 (21.5)**	**10.7 (11.5)**	**3.8 (6.5)**	**95.9**
cvi42	54.3 (40.9)	47.8 (37.8)	15.8 (17.8)	5.6 (9.0)	31.9
U-Net	77.7 (27.1)	69.6 (27.9)	15.1 (19.3)	5.7 (14.2)	84.2
U-Net-SAD	81.8 (22.5)	73.7 (23.7)	12.3 (14.1)	4.1 (7.1)	93.4
RVES					
FCN	74.7 (24.5)	64.4 (24.9)	13.6 (19.9)	6.1 (16.3)	87.6
FCN-SAD	**79.2 (20.1)**	**69.1 (21.6)**	**11.2 (12.5)**	**4.1 (7.6)**	**93.3**
cvi42	53.7 (38.0)	45.5 (34.0)	12.9 (12.5)	4.7 (5.8)	64.3
U-Net	71.3 (28.1)	61.4 (27.7)	14.6 (19.0)	6.1 (16.0)	88.4
U-Net-SAD	74.8 (24.8)	64.6 (24.9)	12.1 (13.0)	4.2 (6.6)	88.7

The Dice metrics for FCN method were $86.5\mathrm{\%}$, $83.2\mathrm{\%}$, $80.3\mathrm{\%}$ and $74.7\mathrm{\%}$ for LVED, LVES, RVED and RVES, respectively. The corresponding Dice metrics for FCN-SAD method were $90.6\mathrm{\%}$, $85.0\mathrm{\%}$, $84.4\mathrm{\%}$ and $79.2\mathrm{\%}$, respectively.

Sensitivity, specificity, positive predictive value (PPV) and negative predictive value (NPV) are summarized in Table 4.

**Table 4:** Mean (SD) of different statistical metrics for nearest-neighbor down-sampling

	Sensitivity (%)	Specificity (%)	PPV (%)	NPV (%)
LVED				
FCN	85.7 (22.7)	99.9 (0.4)	89.5 (21.9)	99.8 (0.3)
FCN-SAD	**91.7 (13.4)**	99.9 (0.2)	**90.9 (15.5)**	**99.9 (0.2)**
cvi42	79.5 (35.7)	99.6 (0.7)	69.8 (34.2)	99.7 (0.6)
U-Net	83.0 (25.9)	**99.9 (0.1)**	89.7 (21.9)	99.8 (0.3)
U-Net-SAD	86.8 (22.2)	99.9 (0.2)	89.2 (22.0)	99.8 (0.2)
LVES				
FCN	82.9 (22.7)	99.9 (0.2)	86.4 (22.4)	99.8 (0.2)
FCN-SAD	**88.5 (19.0)**	99.9 (0.3)	84.7 (20.8)	**99.9 (0.1)**
cvi42	79.6 (33.8)	99.7 (0.6)	67.0 (32.5)	99.8 (0.3)
U-Net	79.0 (27.5)	**99.9 (0.2)**	84.1 (26.0)	99.8 (0.2)
U-Net-SAD	82.1 (23.4)	99.9 (0.2)	**87.1 (20.6)**	99.8 (0.2)
RVED				
FCN	79.6 (25.4)	99.7 (0.3)	84.4 (22.6)	99.6 (0.6)
FCN-SAD	**84.9 (21.5)**	99.7 (0.4)	86.2 (20.3)	**99.7 (0.4)**
cvi42	53.1 (41.3)	99.7 (0.5)	60.2 (42.4)	99.0 (1.1)
U-Net	77.0 (29.4)	99.7 (0.4)	84.1 (23.5)	99.6 (0.6)
U-Net-SAD	81.0 (24.6)	**99.8 (0.3)**	**86.3 (20.6)**	99.6 (0.5)
RVES				
FCN	75.0 (26.6)	99.7 (0.4)	79.5 (25.1)	99.7 (0.3)
FCN-SAD	**83.5 (20.0)**	99.6 (0.6)	79.0 (23.0)	**99.8 (0.2)**
cvi42	54.3 (39.5)	99.7 (0.5)	57.3 (39.0)	99.4 (0.7)
U-Net	70.8 (30.3)	99.8 (0.4)	77.7 (27.8)	99.7 (0.4)
U-Net-SAD	74.2 (27.1)	**99.8 (0.3)**	**80.3 (24.3)**	99.7 (0.3)

For both methods, average absolute and average relative deviation of the automatically segmented volumes from manually-segmented volumes, stroke volumes and ejection fractions are reported in Table 5. A smaller deviation indicates better conformity between automatically- and manually derived contours.

**Table 5:** Mean (SD) of the volume/stroke volume (SV)/ejection fraction (EF) differences between predicted and manual segmentations for nearest-neighbor down-sampling

	FCN	FCN-SAD	cvi42	U-Net	U-Net-SAD
Absolute difference					
LVEDV (mL)	4.6 (4.0)	**2.9 (3.1)**	12.8 (18.8)	8.3 (9.3)	5.3 (4.7)
LVESV (mL)	3.6 (3.9)	**2.7 (2.5**)	6.3 (7.3)	5.3 (6.5)	3.9 (3.9)
RVEDV (mL)	12.0 (18.7)	**7.7 (9.8)**	30.3 (40.6)	12.6 (20.7)	9.4 (12.6)
RVESV (mL)	6.7 (8.3)	**5.4 (5.6)**	9.8 (15.1)	6.7 (7.7)	5.7 (8.3)
LVSV (mL)	3.7 (3.6)	**2.2 (1.8)**	10.5 (17.7)	4.1 (4.7)	3.0 (2.8)
RVSV (mL)	9.6 (13.1)	**6.0 (6.8)**	22.4 (28.3)	10.6 (15.8)	6.9 (7.6)
LVEF (%)	4.1 (4.9)	**2.8 (1.9)**	107.8 (613.3)	4.8 (6.5)	5.4 (14.1)
RVEF (%)	4.1 (3.0)	3.7 (3.0)	48.5 (193.0)	4.5 (4.8)	**3.5 (3.3)**
Relative difference					
LVEDV (%)	7.1 (11.4)	**4.0 (5.7)**	21.1 (32.5)	10.7 (16.4)	8.1 (15.6)
LVESV (%)	12.8 (14.1)	**9.5 (7.6)**	28.9 (56.9)	17.1 (19.5)	13.5 (16.9)
RVEDV (%)	10.4 (14.1)	**7.4 (8.7)**	27.0 (28.5)	12.1 (18.2)	8.8 (10.7)
RVESV (%)	13.9 (15.6)	12.5 (12.4)	24.9 (28.4)	14.7 (16.3)	**11.2 (11.6)**
LVSV (%)	8.8 (13.4)	**5.1 (6.8)**	26.7 (35.2)	9.9 (16.6)	8.3 (16.1)
RVSV (%)	13.5 (14.3)	**9.4 (9.0)**	32.6 (32.5)	15.8 (20.0)	11.1 (12.1)
LVEF (%)	6.9 (8.1)	**4.6 (2.9)**	182.2 (1036.0)	7.8 (10.8)	9.2 (23.8)
RVEF (%)	7.1 (5.3)	6.4 (5.2)	89.3 (366.3)	7.4 (7.5)	**5.7 (5.0)**

The ranges of LV end-diastolic volume (LVEDV), LV end-systolic volume (LVESV), LV stroke volume (LVSV) and LV ejection fraction (LVEF) for the $38$ test subjects were ($10\,\mathrm{mL}$ to $202\;\mathrm{mL}$), ($4\;\mathrm{mL}$ to $91\;\mathrm{mL}$), ($6\;\mathrm{mL}$ to $128\;\mathrm{mL}$) and ($30\mathrm{\%}$ to $75\mathrm{\%}$), respectively. The ranges of RV end-diastolic volume (RVEDV), end-systolic volume (RVESV), stroke volume (RVSV) and ejection fraction (RVEF) for the $38$ test subjects were ($20\;\mathrm{mL}$ to $265\;\mathrm{mL}$), ($6\;\mathrm{mL}$ to $130\;\mathrm{mL}$), ($12\;\mathrm{mL}$ to $138\;\mathrm{mL}$) and ($32\mathrm{\%}$ to $84\mathrm{\%}$), respectively.

The p-values for the paired sample t-test of LVEDV, LVESV, RVEDV and RVESV to test the null hypothesis that predicted and ground-truth volumes have identical expected values are tabulated in Table 6. A p-value greater than $0.05$ is considered as passing the t-test and is boldfaced in Table 6. The ICC values for the paired predicted and ground-truth values of LVEDV, LVESV, RVEDV and RVESV are listed in Table 6. An ICC value greater than $0.90$ is considered as an excellent agreement and is boldfaced in Table 6.

**Table 6:** ICC and P-values of the paired sample t-test for models trained on nearest-neighbor down-sampled data

	FCN	FCN-SAD	cvi42	U-Net	U-Net-SAD
P-value					
LVEDV	7.428*e*−6	4.438*e*−3	**0.289**	1.278*e*−4	9.886*e*−4
LVESV	1.440*e*−4	**0.397**	0.015	1.284*e*−3	8.218*e*−4
RVEDV	0.01	**0.123**	5.181*e*−5	3.026*e*−2	4.666*e*−3
RVESV	**0.054**	**0.548**	3.912*e*−3	5.535*e*−4	4.136*e*−3
ICC					
LVEDV	**0.992**	**0.996**	0.893	**0.964**	**0.989**
LVESV	**0.962**	**0.982**	0.893	0.896	**0.957**
RVEDV	**0.931**	**0.979**	0.643	**0.921**	**0.964**
RVESV	**0.936**	**0.967**	0.819	**0.937**	**0.936**

**Fig. 6:**
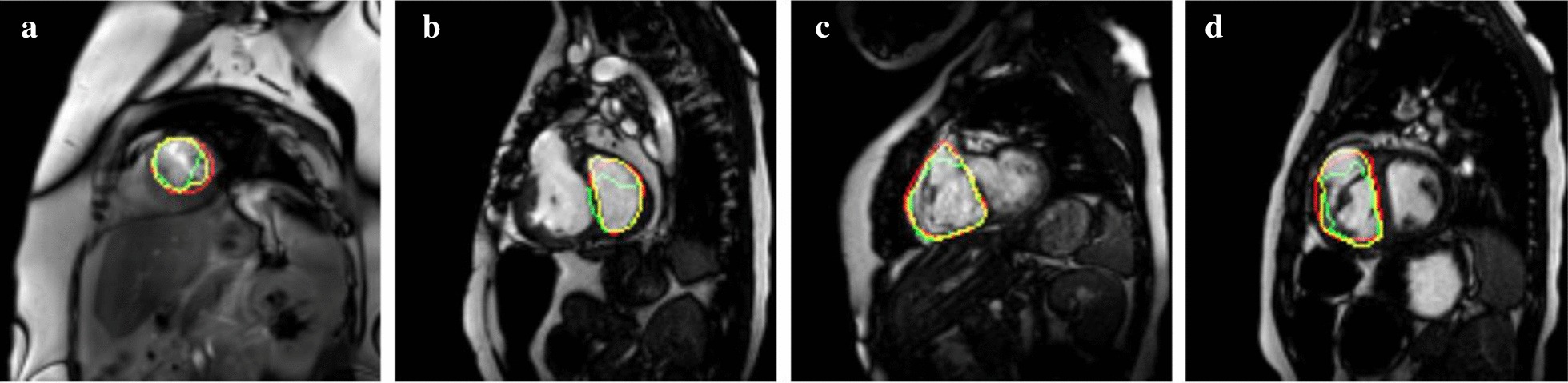
Sample segmentation. **a** LV end-diastole (LVED), **b** LV end-systole (LVES), **c** RV end-diastole (RVED), **d** RV end-systole (RVES)

Exemplary LV and RV segmentations at ES and ED are shown in Fig. 6. Red contours correspond to the ground-truth (i.e., manual annotation) whereas green and yellow contours correspond to the predicted delineations by FCN and FCN-SAD methods, respectively.

**Fig. 7:**
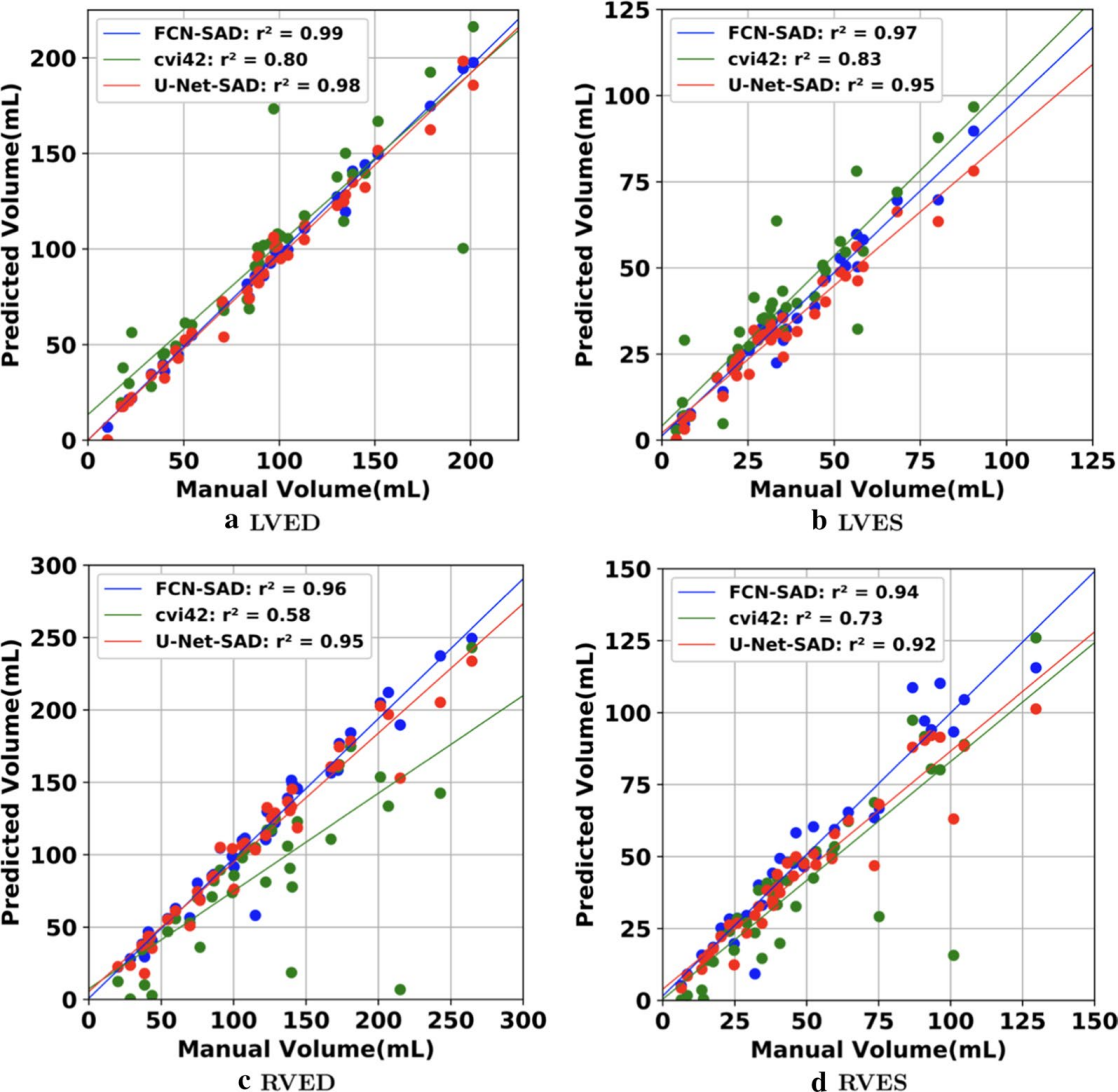
Correlation plots for nearest-neighbor down-sampling. **a** LV end-diastolic volume (LVEDV) , **b** LV end-systolic volume (LVESV) , **c** RV end-diastolic volume (RVEDV) , **d** RV end-systolic volume (RVESV)

**Fig. 8:**
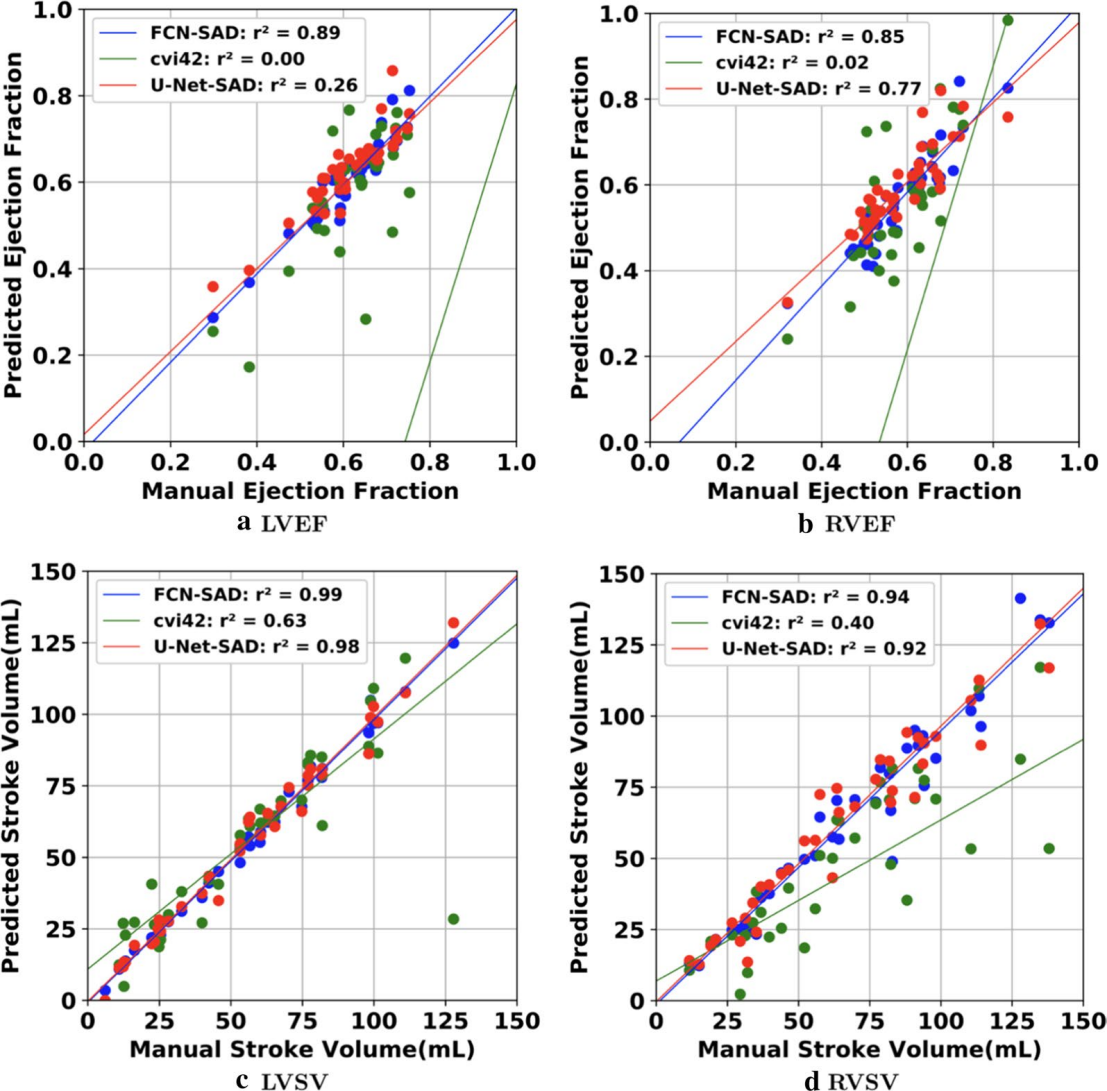
Correlation plots for nearest-neighbor down-sampling. **a** LV ejection fraction (LVEF), **b** RV ejection fraction (RVEF), **c** LV stroke volume (LVSV) , **d** RV stroke volume (RVSV)

**Fig. 9:**
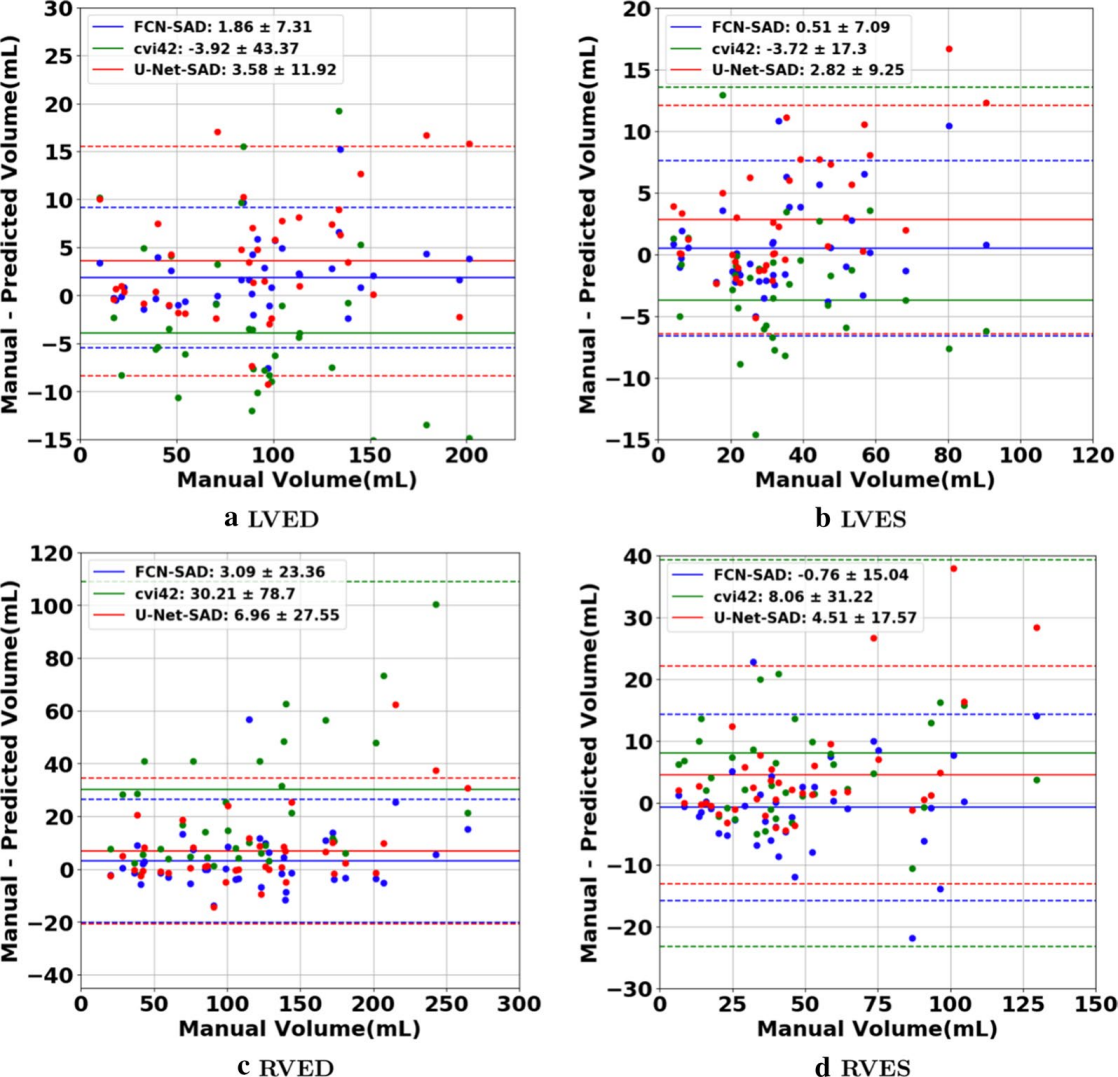
Bland–Altman plots for nearest-neighbor down-sampling. **a** LVEDV, **b** LVESV, **c** RVEDV, **d** RVESV

**Fig. 10:**
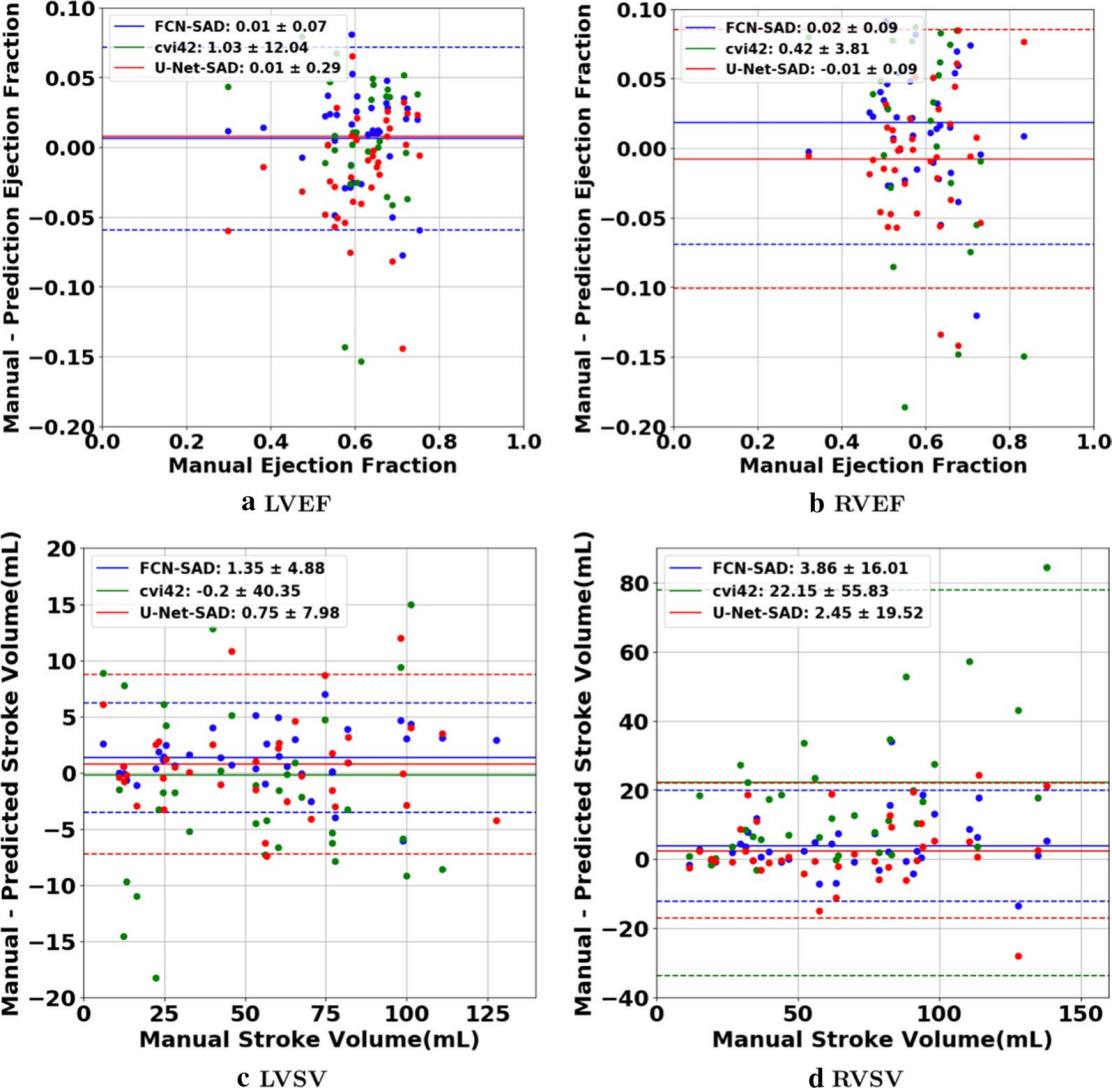
Bland–Altman plots for nearest-neighbor down-sampling. **a** LVEF, **b** RVEF, **c** LVSV, **d** RVSV

The correlation and Bland–Altman plots are shown in Figs. 7, 8, 9 and 10. The FCN-SAD results are depicted by blue dots. As shown in Figs. 7 and 8, the points deviated from the line $y=x$ are due to the mismatch between prediction and ground-truth. The Bland–Altman diagrams are commonly used to evaluate the agreement among clinical measures and identifying any systematic difference (i.e., fixed bias, outliers etc.). The bias values of the FCN for LVEDV, LVESV, RVEDV and RVESV were $3.9\mathrm{mL}$, $3.0\mathrm{mL}$, $8.9\mathrm{mL}$ and $3.3\mathrm{mL}$, respectively, whereas the bias values of the FCN-SAD for LVEDV, LVESV, RVEDV and RVESV were $1.9\mathrm{mL}$, $0.5\mathrm{mL}$, $3.1\mathrm{mL}$ and $-0.8\mathrm{mL}$, respectively. The $95\mathrm{\%}$ confidence interval of difference between automatic segmentation and ground-truth is shown as dashed lines representing $\pm 1.96$ standard deviation.

#### Segmentation performance for bi-cubical down-sampling

The results for the bi-cubical down-sampling method are reported in Table 7. FCN-SAD method’s Dice metrics for LVED, LVES, RVED and RVES were $91.0\mathrm{\%}$, $86.8\mathrm{\%}$, $84.7\mathrm{\%}$ and $80.6\mathrm{\%}$, respectively. The FCN-SAD’s t-test p-values for LVED, LVES, RVED and RVES are $0.27$, $0.09$, $0.08$, and $0.66$, respectively. FCN-SAD method unequivocally passes the paired sample t-test for LV and RV at both ED and ES phases.

**Table 7:** Different quantitative metrics for models trained on bi-cubically down-sampled data

	Dice (%)	Jaccard (%)	Rel. volume difference (%)	${\text{R}}_{{{\text{vol}}}}^{2} (\%)$	t-test p-value
LVED					
FCN	88.5 (18.4)	82.6 (20.3)	6.0 (10.3)	98.5	0.0147
FCN-SAD	**91.0 (14.9)**	**85.8 (17.0)**	**4.6 (6.1)**	**99.2**	**0.2739**
U-Net	85.5 (23.0)	79.5 (24.0)	10.7 (17.7)	89.8	4.755*e*−3
U-Net-SAD	87.4 (21.1)	81.7 (22.3)	8.3 (15.9)	97.7	0.443*e*−3
LVES					
FCN	83.1 (22.6)	75.6 (23.8)	10.1 (9.3)	95.7	**0.0786**
FCN-SAD	**86.8 (16.5)**	**79.4 (18.9)**	**7.9 (5.1)**	**97.8**	**0.0945**
U-Net	81.6 (22.6)	73.4 (23.8)	23.4 (39.9)	79.1	9.913*e*−4
U-Net-SAD	83.9 (20.7)	76.1 (22.0)	15.6 (27.0)	93.3	3.786*e*−4
RVED					
FCN	80.9 (22.9)	72.6 (24.3)	9.3 (14.2)	87.7	0.0159
FCN-SAD	**84.7 (18.8)**	**76.8 (20.8)**	**6.8 (8.6)**	**94.9**	**0.0858**
U-Net	76.5 (29.5)	69.0 (29.6)	12.4 (17.9)	81.8	0.0134
U-Net-SAD	81.8 (22.8)	73.8 (24.4)	9.7 (12.4)	91.8	0.0251
RVES					
FCN	77.2 (22.8)	67.2 (23.6)	13.6 (15.6)	90.6	0.0226
FCN-SAD	**80.6 (19.7)**	**70.9 (21.2)**	**11.0 (13.8)**	**92.9**	**0.6585**
U-Net	70.2 (30.4)	60.9 (29.4)	18.5 (19.9)	82.6	0.151*e*−3
U-Net-SAD	74.8 (25.5)	64.9 (25.6)	13.8 (15.4)	88.1	1.783*e*−3

**Fig. 11:**
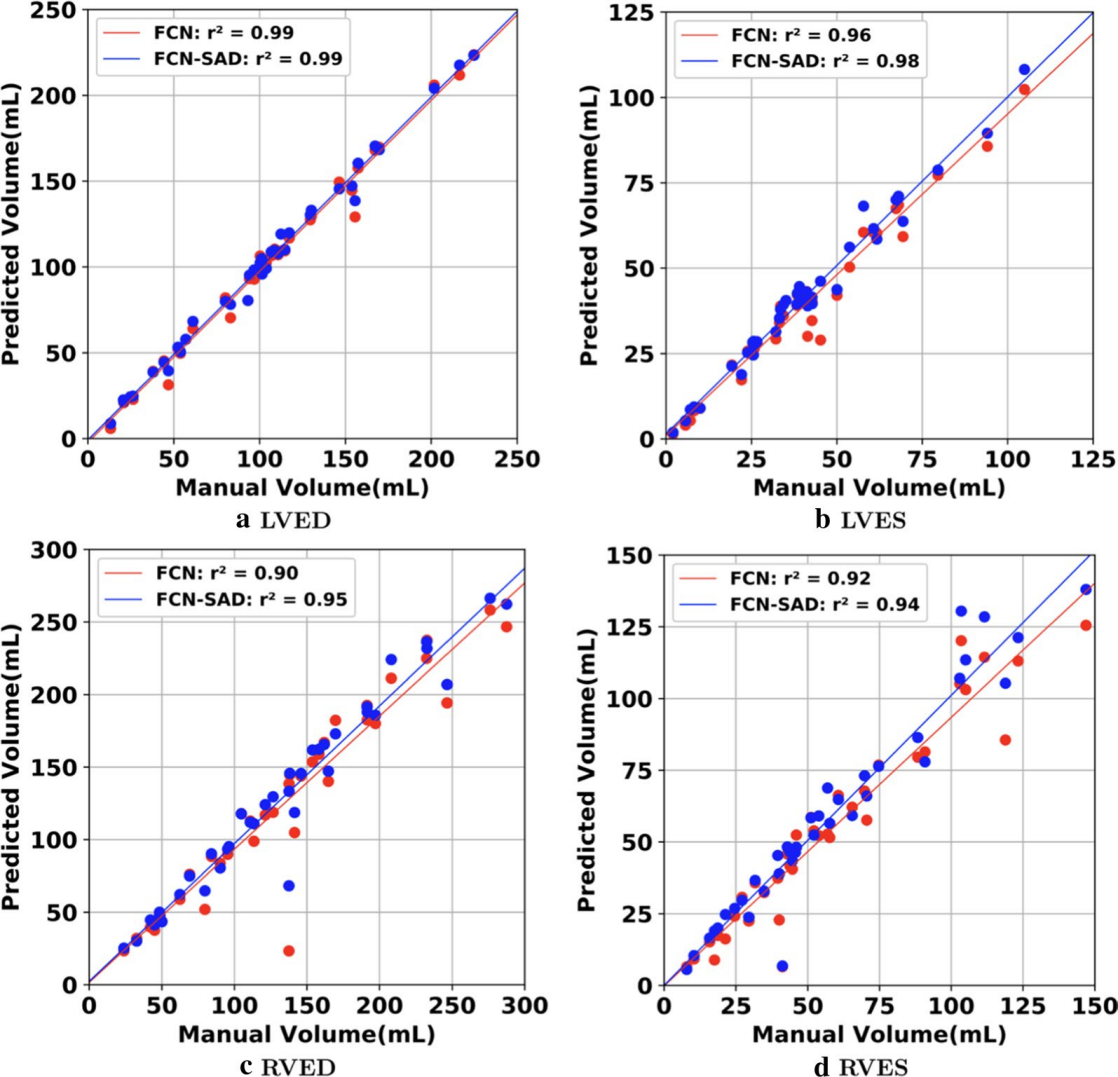
Correlation plots for bi-cubical down-sampling. **a** LVEDV, **b** LVESV, **c** RVEDV, **d** RVESV

**Fig. 12:**
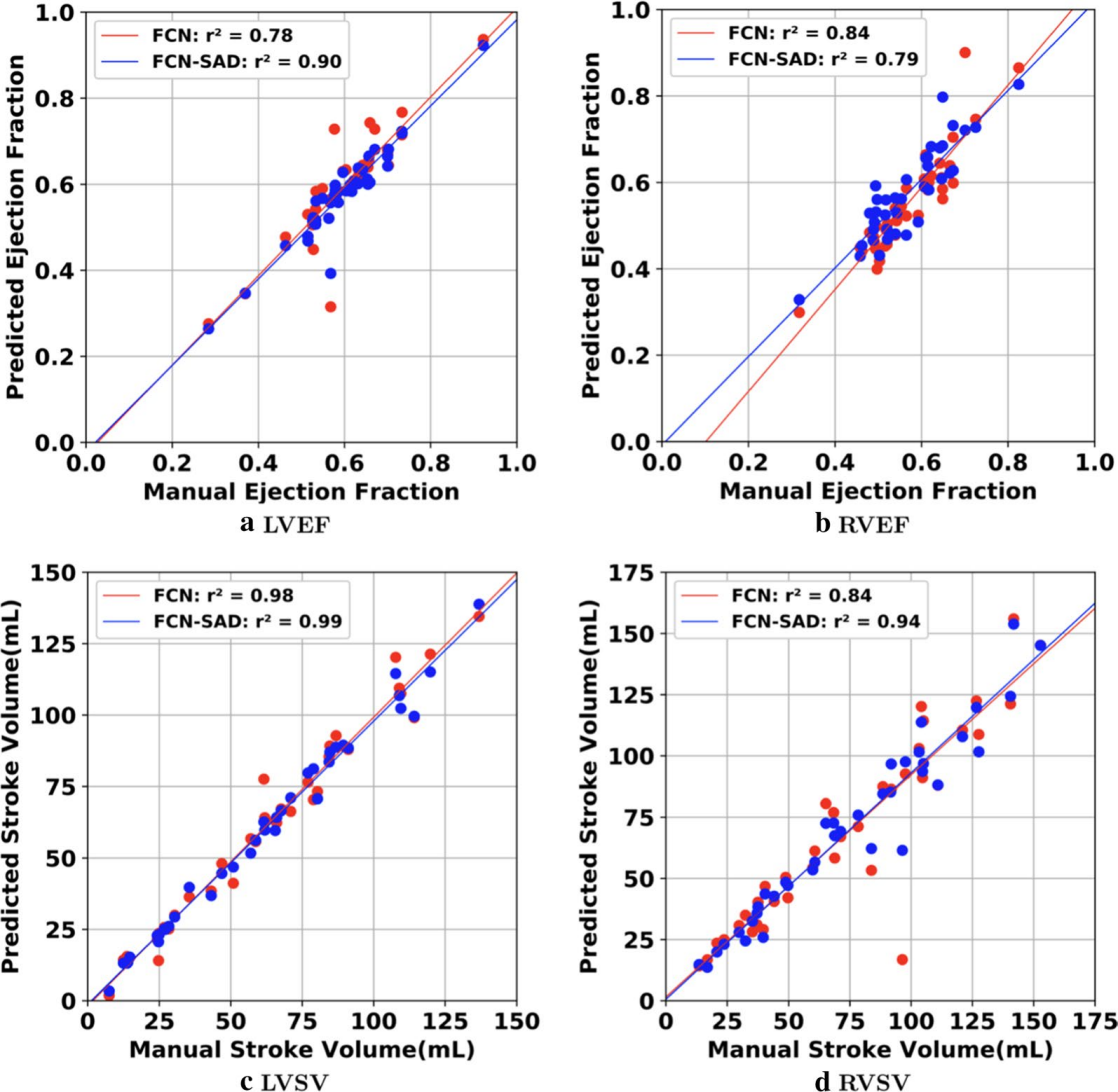
Correlation plots for bi-cubical down-sampling. **a** LVEF, **b** RVEF, **c** LVSV, **d** RVSV

**Fig. 13:**
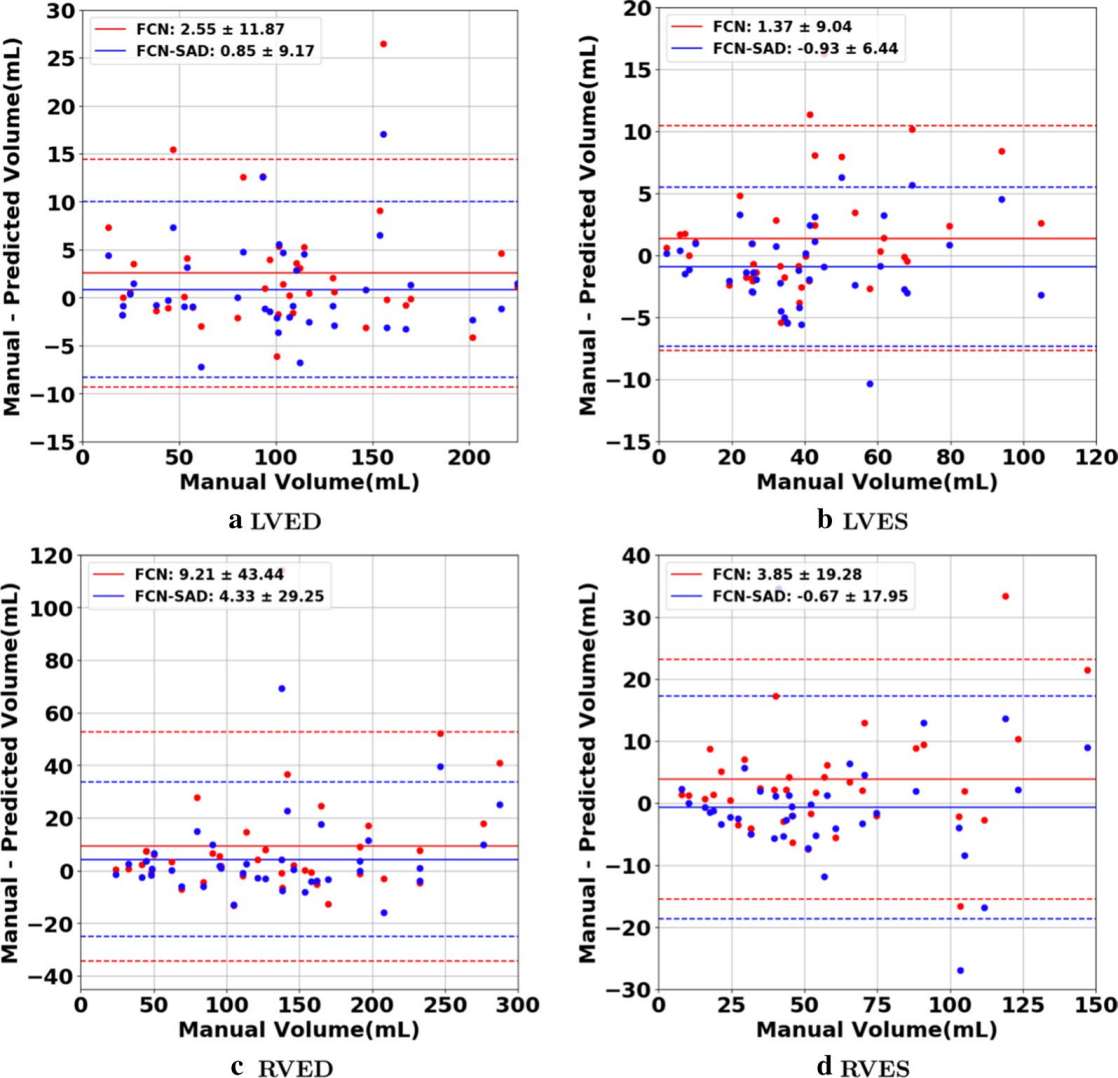
Bland–Altman plots for bi-cubical down-sampling. **a** LVEDV, **b** LVESV, **c** RVEDV, **d** RVESV

**Fig. 14:**
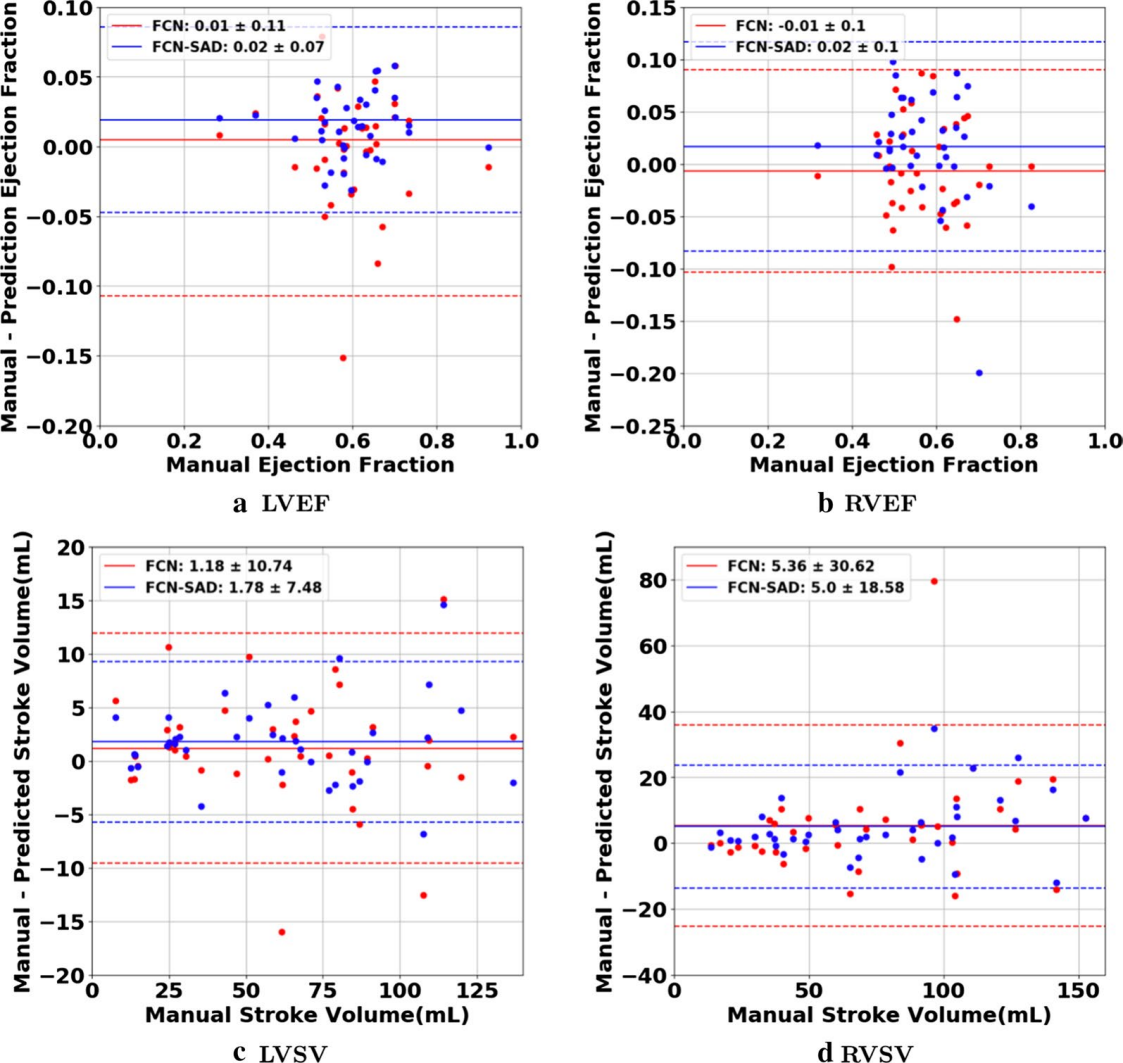
Bland–Altman plots for bi-cubical down-sampling. **a** LVEF, **b** RVEF, **c** LVSV, **d** RVSV

The correlation and Bland–Altman plots for ES and ED ventricular volumes, ejection fractions and stroke volumes for the bi-cubical down-sampling method are depicted in Figs. 11, 12, 13 and 14.

#### Segmentation performance for cvi42

The cvi42-associated Dice metrics were $73.2\mathrm{\%}$, $71.0\mathrm{\%}$, $54.3\mathrm{\%}$ and $53.7\mathrm{\%}$ for LVED, LVES, RVED and RVES, respectively. The corresponding sensitivity, specificity, PPV and NPV are summarized in Table 4. The absolute and relative deviations of automatically- from manually-segmented results for LV and RV volumes at ED and ES as well as SV and EF are summarized in the third column of Table 5.

The correlation and Bland–Altman plots for cvi42 are shown by green dots in Figs. 7, 8, 9 and 10. The bias values of the cvi42 for LVEDV, LVESV, RVEDV and RVESV were $-3.9\,\mathrm{mL}$, $-3.7\,\mathrm{mL}$, $30.2\,\mathrm{mL}$ and $8.1\,\mathrm{mL}$, respectively.

#### Segmentation performance for U-Net with nearest-neighbor down-sampling

Simulations were carried out on the images that were down-sampled using nearest-neighbor method. The average Dice metric, Jaccard index, Hausdorff distance, mean contour distance, and ${R}_{vol}^{2}$ for U-Net and U-Net-SAD computed based on the ground-truth are reported in Table 3.

The Dice metrics for U-Net method were $84.5\mathrm{\%}$, $79.4\mathrm{\%}$, $77.7\mathrm{\%}$ and $71.3\mathrm{\%}$ for LVED, LVES, RVED and RVES, respectively. The corresponding Dice metrics for U-Net-SAD method were $87.1\mathrm{\%}$, $82.3\mathrm{\%}$, $81.8\mathrm{\%}$ and $74.8\mathrm{\%}$, respectively.

Sensitivity, specificity, PPV and NPV for U-Net and U-Net-SAD are summarized in Table 4.

The absolute and relative difference between predicted and ground-truth volumes for LV and RV chambers at ED and ES as well as SV and EF are summarized in the last two columns of the Table 5.

The correlation and Bland–Altman plots for U-Net-SAD are shown by red dots in Figs. 7, 8, 9 and 10. The bias values of the U-Net for LVEDV, LVESV, RVEDV and RVESV were $7.2\mathrm{mL}$, $4.2\mathrm{mL}$, $8.4\mathrm{mL}$ and $5.4\mathrm{mL}$, respectively. The corresponding bias values of U-Net-SAD for LVEDV, LVESV, RVEDV and RVESV were $3.6\mathrm{mL}$, $2.8\mathrm{mL}$, 7.0 $\mathrm{mL}$, and $4.5\mathrm{mL}$, respectively.

#### Segmentation performance for U-Net with bi-cubical down-sampling

Using the images that were down-sampled according to the bi-cubical method, the average Dice metric, Jaccard index, relative volume difference and ${R}_{vol}^{2}$ for U-Net and U-Net-SAD calculated based on the ground-truth are reported in Table 7.

The Dice metrics for U-Net method were $85.5\mathrm{\%}$, $81.6\mathrm{\%}$, $76.5\mathrm{\%}$ and $70.2\mathrm{\%}$ for LVED, LVES, RVED and RVES, respectively. The corresponding Dice metrics for U-Net-SAD method were $87.4\mathrm{\%}$, $83.9\mathrm{\%}$, $81.8\mathrm{\%}$, and $74.8\mathrm{\%}$, respectively.

#### Segmentation performance for FCN-2.0 and FCN-SAD-2.0

To avoid conflict with the definition of HD, MCD, etc., CMR images with no ground-truth segmentation contours are removed from the test set. The average Dice metric, Jaccard index, Hausdorff and mean contour distance for FCN-2.0 and FCN-SAD-2.0 are reported in Table 8. The Dice metrics for FCN-2.0 were $86.7\mathrm{\%}$, $82.8\mathrm{\%}$, $80.8\mathrm{\%}$ and $72.4\mathrm{\%}$ for LVED, LVES, RVED and RVES, respectively. The corresponding Dice metrics for FCN-SAD-2.0 were $91.3\mathrm{\%}$, $86.7\mathrm{\%}$, $84.5\mathrm{\%}$ and $77.0\mathrm{\%}$ for LVED, LVES, RVED and RVES, respectively.

**Table 8:** Mean (SD) of different quantitative metrics for nearest-neighbor down-sampling (CHD + ACDC datasets)

	Dice (%)	Jaccard (%)	HD (mm)	MCD (mm)
LVED				
FCN-2.0	86.7 (22.7)	81.1 (23.7)	7.1 (13.0)	3.1 (10.4)
FCN-SAD-2.0	**91.3 (15.1)**	**86.2 (16.6)**	**5.2 (9.1)**	**2.0 (7.8)**
LVES				
FCN-2.0	82.8 (23.1)	75.3 (24.2)	8.3 (17.8)	3.6 (12.4)
FCN-SAD-2.0	**86.7 (17.6)**	**79.6 (19.6)**	**6.0 (10.9)**	**2.7 (10.0)**
RVED				
FCN-2.0	80.8 (22.7)	72.3 (24.0)	14.3 (18.9)	5.8 (14.7)
FCN-SAD-2.0	**84.5 (18.8)**	**76.5 (20.6)**	**12.1 (16.0)**	**4.2 (9.1)**
RVES				
FCN-2.0	72.4 (26.8)	62.2 (26.5)	15.8 (21.3)	7.4 (17.9)
FCN-SAD-2.0	**77.0 (22.8)**	**66.9 (23.7)**	**13.4 (16.4)**	**4.6 (8.6)**

## Discussion

Many challenges currently exist for segmenting cardiac chambers from CMR images, notably in pediatric and CHD patients [12, 28–30]. In the past few years, a great deal of activities involved CMR segmentation using the learning-based approaches [5–8]. Despite their relative successes, they still have certain limitations. Small datasets incur a large bias to the segmentation, which makes these methods unreliable when the heart shape is outside the learning set (e.g., CHDs and post-surgically remodeled hearts). In brief, in pediatric cardiac imaging, learning-based methods remain computationally difficult and their predictive performance are less than optimal, due to the complexity of estimating parameters, as their convergence is not guaranteed [31].

While traditional deep-learning methods achieve good results for subjects with relatively normal structure, they are not as reliable for segmenting the CMR images of CHD patients [7, 8]. It is believed that the absence of large databases that include CMR studies from heterogeneous CHD subjects significantly limits the performance of these traditional models [32]. To address this shortcoming, our new method simultaneously generates synthetic CMR and their corresponding segmented images. Our DCGAN-based FCN model was tested on a heterogeneous dataset of pediatric patients with complex CHDs.

Current software platforms designed for adult patients, such as cvi42 by Circle Cardiovascular Imaging Inc, were previously reported to have many shortcomings when used for pediatric or CHD applications. Children are not scaled little adults; pediatric patient characteristics, such as cardiac anatomy, function, higher heart rates, degree of cooperativity, and smaller body size, all affect post-processing approaches to CMR, and there is currently no CMR segmentation tool dedicated to pediatric patients. Our major motivation for this study was the fact that current clinically available segmentation tools cannot be reliably used for children.

The LV and RV volumes were computed using our automatic segmentation methods, U-Net model and the cvi42 (version 5.10.1.) were compared with the ground-truth volumes. As reported in Table 5, cvi42′s rendered volumes led to a significant difference between the predicted and true values of volumetric measures although it uses the original high quality and high resolution CMR images coming from the scanner for its predictions. Synthetic data augmentation also improved volume prediction for the U-Net. In addition, as shown in Table 5, FCN-SAD method outperforms U-Net-SAD for both chambers at end-systole and end-diastole. As reported in Table 7, our FCN-SAD passed the t-test’s null hypothesis that the predicted and ground-truth volumes have identical expected values for LVED, LVES, RVED and RVES. However, cvi42 only passed the t-test for LVED. Since the p-value is largely affected by the sample size etc., the ICC values are also reported for all models in Table 6. Our FCN and FCN-SAD models led to an excellent correlation coefficient for both LV and RV at ED and ES. U-Net-SAD also resulted in ICC values greater than $0.90$; however, U-Net failed to achieve the excellent threshold for LVES. All cvi42′s ICC values are below the excellent threshold as well. Although the exact deep learning architecture of cvi42 is not known to us, in our opinion, the main reason for the relatively poor performance of cvi42 on pediatric CHD patients is the training of its neural network on the UK Biobank (as declared on their website), which is limited to the adult CMR images. More precisely, UK Biobank dataset does not represent features that are inherent to the heart of children with CHD.

As indicated in Tables 3 and 4, our method outperforms cvi42 in Dice metric, Jaccard index, HD, MCD, volume correlation, sensitivity, specificity, PPV and NPV. For LV segmentation, FCN-SAD improved Dice metric from $73.2\mathrm{\%}$ to $90.6\mathrm{\%}$ and from $71.0\mathrm{\%}$ to $85.0\mathrm{\%}$ over cvi42 at end-diastole and end-systole, respectively. Similar improvement was observed for RV segmentation where Dice metric was improved from $54.3\mathrm{\%}$ to $84.4\mathrm{\%}$ and from $53.7\mathrm{\%}$ to $79.2\mathrm{\%}$ at end-diastole and end-systole, respectively. FCN-SAD also reduced the average Hausdorff and mean contour distances compared to cvi42, which improved alignment between the contours as observed for both LV and RV at ED and ES. Similar improvement was observed for FCN-SAD over U-Net-SAD. For LV segmentation, FCN-SAD improved the Dice metric over U-Net-SAD from $87.1\mathrm{\%}$ to $90.6\mathrm{\%}$ for ED, and from $82.3\mathrm{\%}$ to $85.0\mathrm{\%}$ for ES. Similarly, FCN-SAD improved U-Net-SAD for RV segmentation from $81.8\mathrm{\%}$ to $84.4\mathrm{\%}$ for ED, and from $74.8\mathrm{\%}$ to $79.2\mathrm{\%}$ for ES. FCN-SAD also led to lower HD and MCD values compared to the U-Net-SAD method.

The data augmentation using DCGAN improved the Dice metric values by about $3\mathrm{\%}$ in FCN-SAD compared to our FCN method. Improvement was observed for Jaccard index, HD, MCD, volume correlation, sensitivity, specificity, PPV and NPV as well.

As shown in Table 3, synthetic data augmentation improved both Dice and Jaccard indices by about $3\mathrm{\%}$ for U-Net, which shows that synthetic data augmentation can improve the performance of FCN methods regardless of the type. Compared to the U-Net method, similar improvement was observed in U-Net-SAD for both HD and MCD as well. Table 3 reveals that our FCN method outperforms U-Net. Similarly, our FCN-SAD method outperforms U-Net-SAD in all metrics for LVED, LVES, RVED and RVES.

Synthetic data augmentation also improved both Dice and Jaccard indices by about $4\mathrm{\%}$ for FCN-2.0. Similar improvement was observed in FCN-SAD-2.0 for both HD and MCD, which indicates better alignment between predicted and manual segmentation contours.

As expected, for all methods, RV segmentation proved to be more challenging than LV segmentation due to the complex RV shape and anatomy. The sophisticated crescent shape of RV as well as the considerable variations among the CHD subjects make it harder for the segmentation models to learn the mapping from a CMR image to its corresponding mask. Another major limiting factor that affects the performance of RV segmentation is the similarity of the signal intensities for RV trabeculations and myocardium.

Our methodology has overcome some of these limiting issues by learning the generative process through which each RV chamber is segmented. This information is then passed to the segmentation model via synthetic samples obtained from that generative process.

Corroborating the fact suggested by Yu et al., [33], larger contours can be more precisely delineated compared to the smaller ones. Segmentation of the CMR slices near the apex, particularly at the end-systole, is more challenging due to their small and irregular shape. Table 3 shows that both Dice and Jaccard indices are higher at ED versus ES for both ventricles. Another possible reason for lower performance at ES could be attributed to their small mask area and the smaller values of denominator at Eq. (3), which can lead to a major effect on the final values of these metrics, in case of even a few misclassified pixels.

Figures 7a and b show that the results generated by our FCN-SAD model leads to high correlation for LVEDV and LVESV. This in turn leads to high correlation in EF and SV as shown in Figs. 8a and c in addition to ${R}_{vol}^{2}$ values in Table 3. Similarly, a high correlation was observed for RVEDV and RVESV in Figs. 7c and d, which subsequently leads to high correlation in EF and SV as shown in Figs. 8b and d as well as the ${R}_{vol}^{2}$ scores in Table 3. Bland–Altman analyses in Figs. 9 and 10 show negligible bias for the results due to FCN-SAD model trained on the synthetically augmented data. Bland–Altman plots show that applying the FCN-SAD method reduced the mean and standard deviation of error in predicted volumes and tightened the confidence interval compared to other methods.

The average elapsed times to segment a typical image in our GPU-accelerated computing platform is $10\mathrm{ms}$. Overall, our model takes $0.1\mathrm{s}$ to process each patient’s CMR data. Simulations show that even on a common CPU-based computing platform, our method requires about $1.3\mathrm{s}$ to segment each patient’s CMR images, which indicates the clinical applicability of our automated segmentation model.

Similar quantitative and volumetric results were observed when the whole training and validation procedures were repeated with a different random split of training and test subjects. This indicates that no noticeable bias has occurred by the way subjects are categorized into training and test set.

Finally, we would like to emphasize on the significance of the choice of down-sampling method over the segmentation performance. The entire process of training and testing was repeated using both nearest-neighbor and bi-cubical down-sampling methods. Compared to the nearest-neighbor down-sampling method, the bi-cubical down-sampling provides a better performance for almost all studied models, except for the segmentation of the RVED using U-Net and U-Net-SAD. For example, the bi-cubical FCN-SAD results unequivocally passed the t-test for all chambers denoting the predicted and ground-truth volumes have identical expected value for LVED while the nearest-neighbor FCN-SAD did not. In our opinion, the main reason behind the superior performance of the bi-cubical down-sampling method is its larger mask area compared to the nearest-neighbor method.

### Limitations

As a limitation, our method applied to the CMR datasets of patients with two ventricles, and was not yet trained to analyze patients with a systemic RV. Overall, to the computer, CMR images of hypoplastic left heart syndrome hearts are considered totally different objects. Therefore, a new training algorithm is needed to analyze the single ventricle hearts. We are currently designing a new model for that, which is beyond the scope of the present work. A second limitation of our method is that it must be calibrated before it can be applied to CMR images acquired from another scanner and with different cohort characteristics.

It should also be mentioned that we have used Fréchet Inception Distance (FID) to discriminate between real and synthetic CMR images. While the FID is commonly used, human judgment is still the best measure, although it is subjective and depends upon the experience. To derive a statistically significant validation, a large cohort of imaging physicians are needed which we aim to accomplish in near future.

We used OsiriX Lite software to calculate the volumes; however, OsiriX Lite may underestimate the volume if one image slice has no predicted segmentation due to its small chamber size. This was the case for the outliers at the bottom of Figs. 7c and d. Since our dataset did not include epicardial ground-truth contours, the cardiac mass was not calculated. Another limitation of this work is the lack of intra- and inter-observer variability assessments since only one set of manual segmentation was available. Finally, the loss of resolution, caused by the down-sampling, was an inevitable limitation, which led to a compromise among speed, accuracy of the model and the data dimension.

## Conclusions

Manual segmentation is subjective, less reproducible, time consuming and requires dedicated experts. Therefore, fully automated and accurate segmentation methods are desirable to provide precise and reproducible clinical indices such as ventricular ejection fraction, chamber volume, etc. in a clinically actionable time-frame. Our learning-based framework provides an automated, fast, and accurate model for LV and RV segmentation, and its outstanding performance in children with complex CHDs implies its potential to be used in clinics across the pediatric age group.

Contrary to many existing automated approaches, our framework does not make any assumption about the image or the structure of the heart, and performs the segmentation by learning features of the image at different levels of abstraction in the hierarchy of network layers. To improve the robustness and accuracy of our segmentation method, a novel generative adversarial network is introduced to enlarge the training data via synthetically generated and realistic looking samples. The new technique is also applicable on other FCN methods (e.g., U-net) and can improve the FCN performance independent of its specific type. The FCN trained on both real and synthetic data exhibits an improvement in various statistical and clinical measures such as Dice, HD and volume over the existing machine learning methods.

## Data Availability

The CMR datasets analyzed during the current study are available from the public repository at https://github.com/saeedkarimi/Cardiac_MRI_Segmentation

## Appendix: Technical details

### Fully convolutional network architecture

All convolution layers shared the kernel size of $3$, stride of $1$ pixel with hyperbolic tangent function (Tanh) as their activation function. The input for each convolution layer was padded such that the output retains the same length as the original input. To avoid overfitting, ${l}_{2}-$ regularization was applied to control layer parameters during optimization. To circumvent underfitting, a small regularization coefficient of $0.0005$ was selected. These penalties were applied on a per-layer basis and incorporated in the loss function that the network optimizes during training. Each convolution layer’s output was normalized to zero-mean and unit-variance that allows the model to focus on the structural similarities/dissimilarities rather than on the amplitude-driven ones.

The FCN model in Fig. 1 includes approximately $11$ million parameters. Considering our relatively small CMR image dataset of $527$ ($570$) left (right) ventricle images, the network is prone to overfitting. Therefore, in addition to ${l}_{2}-$ regularization, three dropout layers that randomly set $50\mathrm{\%}$ of the input units to $0$ at each update during training were applied after the last convolution layers including $128$, $256$ and $512$ filters.

### Deep convolutional generative adversarial networks

The adversarial modeling framework is comprised of two components, commonly referred to as the generator and discriminator. The functionality of the generator is denoted by a differentiable function, $G$, which maps the input noise variable $z\sim {p}_{Z}\left(z\right)$ to a point $x=G\left(z\right)$ in the data space. The generator should compete against an adversary, i.e., the discriminator, that strives to distinguish between real samples drawn from the genuine CMR data and synthetic samples created by the generator. More precisely, if the functionality of the discriminator is denoted by a differentiable mapping $D$, then $D\left(x\right)$ is a single scalar representing the probability that $x$ comes from the data rather than the generator output. The discriminator is trained to maximize the probability of assigning the correct label to both real and synthetic samples while the generator is simultaneously trained to synthesize samples that the discriminator interprets with high probability as real. More precisely, the discriminator is trained to maximize $D\left(x\right)$ when $x$ is drawn from the data distribution ${p}_{\mathrm{data}}$ while the generator is trained to maximize $D\left(G\left(z\right)\right)$, or equivalently minimize $1-D\left(G\left(z\right)\right)$. Hence, adversarial networks are based on a zero-sum non-cooperative game, i.e., a two-player minimax game in which the generator and discriminator are trained by optimizing the following objective function [34]:

1 $$\underset{G}{\mathit{min}}\;\underset{D}{\mathit{max}}\;{\mathbb{E}}_{x\sim {p}_{data}}\left[\mathit{log}D\left(x\right)\right]+{\mathbb{E}}_{z\sim {p}_{Z}}\left[\mathit{log}\left(1-D\left(G\left(z\right)\right)\right)\right],$$

where ${\mathbb{E}}\left[.\right]$ represents expectation. The adversarial model converges when the generator and discriminator reach a Nash equilibrium, which is the optimal point for the objective function in Eq. (1). Since both $G$ and $D$ strive to undermine each other, a Nash equilibrium is achieved when the generator recovers the underlying data distribution and the output of $D$ is ubiquitously $\frac{1}{2}$, i.e., the discriminator cannot distinguish between real and synthetic data anymore. The optimal generator and discriminator at Nash equilibrium are denoted by ${G}^{*}$ and ${D}^{*}$, respectively. New data samples are generated by feeding random noise samples to the optimal generator ${G}^{*}$.

### DCGAN optimization

The learning rate, parameter ${\beta }_{1}$, and parameter ${\beta }_{2}$ in Adam optimizer were set to $0.0002$, $0.5$, and $0.999$, respectively. The binary cross entropy between the target and the output was minimized. Since Adam, like any other gradient-based optimizer, is a local optimization method, only a local Nash equilibrium can be established between the generator and discriminator. A common method to quantify the quality of the generated synthetic samples is the FID, originally proposed by Heusel et al. [35]. In FID, features of both real and synthetic data are extracted via a specific layer of Inception v3 model [36]. These features are then modeled as multivariate Gaussian, and the estimated mean and covariance parameters are used to calculate the distance as [35]:

2 $$FID\left(s,r\right)={\Vert {\mu }_{s}-{\mu }_{r}\Vert }_{2}^{2}+Tr\left({\Sigma }_{s}+{\Sigma }_{r}-2{\left({\Sigma }_{s}{\Sigma }_{r}\right)}^\frac{1}{2}\right),$$

where $\left({\mu }_{s},{\Sigma }_{s}\right)$ and $\left({\mu }_{r},{\Sigma }_{r}\right)$ are the mean and covariance of the extracted feature from the synthetic and real data, respectively. Lower FID values indicate better image quality and diversity among the set of synthetic samples.

Once the locally optimal generator was obtained, various randomly selected subsets of the generated synthetic images were considered and the one with the lowest FID distance to the set of real samples was chosen.

### Metrics definition

The Dice and Jaccard, as defined in Eq. (3), are two measures of contour overlap with a range from zero to one where a higher index value indicates a better match between the predicted and true contours:

3 $$Dice=\frac{2\left|A\cap B\right|}{\left|A\right|+\left|B\right|} ,\quad Jaccard \,=\,\frac{\left|A\cap B\right|}{\left|A\cup B\right|},$$

where $A$ and $B$ are true and predicted segmentation, respectively. Hausdorff and mean contour distances are two other standard measures that show how far away the predicted and ground-truth contours are from each other. These metrics are defined as:

$$HD=\mathit{max}\left(\underset{a\in \partial A}{\mathit{max}}\;d\left(a,\partial B\right),\underset{b\in \partial B}{\mathit{max}}\;d\left(b,\partial A\right)\right),$$

4 $$MCD=\frac{1}{2\left|\partial A\right|}\sum_{a\in \partial A}d\left(a,\partial B\right)+\frac{1}{2\left|\partial B\right|}\sum_{b\in \partial B}d\left(b,\partial A\right)$$

where $\partial A$ and $\partial B$ denote the contours of the segmentation $A$ and $B$, respectively, and $d\left(a,\partial B\right)$ is the minimum Euclidean distance from point $a$ to contour $\partial B$. The lower values for these metrics indicate better agreement between automated and manual segmentation. The ICC for paired data values $\left({x}_{i},{x}_{i}^{^{\prime}}\right)$, for $i=1,\cdots ,N$, originally proposed in [26], is defined as:

5 $$r=\frac{1}{N{s}^{2}}\sum_{i=1}^{N}\left({x}_{i}-\stackrel{-}{x}\right)\left({x}_{i}^{^{\prime}}-\stackrel{-}{x}\right),$$

where

6 $$\stackrel{-}{x}=\frac{1}{2N}\sum_{i=1}^{N}\left({x}_{i}+{x}_{i}^{^{\prime}}\right),$$

7 $${s}^{2}=\frac{1}{2N}\left(\sum_{i=1}^{N}{\left({x}_{i}-\stackrel{-}{x}\right)}^{2}+\sum_{i=1}^{N}{\left({x}_{i}^{^{\prime}}-\stackrel{-}{x}\right)}^{2}\right).$$

ICC is a descriptive statistic that quantifies the similarity of the samples in the same group.

## Abbreviations

2D: Two dimensional
ACDC: Automated Cardiac Diagnosis Challenge
AI: Artificial intellilgence
bSSFP: Balanced steady state free precesion
CAA: Coronary artery anomaly
CHD: Congenital heart disease
CMR: Cardiovascular magnetic resonance
CNN: Convolutional neural network
CPU: Central processing unit
DCGAN: Deep convolutional generative adversarial network
DORV: Double outlet right ventricle
ED: End-diastole
EF: Ejection fraction
ES: End-systole
FCN: Fully convolutional network
FN: False negative
FP: False positive
GAN: Generative adversarial network
GPU: Graphics processing unit
HD: Hausdorff distance
ICC: Intraclass Correlation Coefficient;
LV: Left ventricle/left ventricular
LVEDV: Left ventricular end-diastolic volume
LVEF: Left ventricular ejection fraction
LVESV: Left ventricular end-systolic volume
LVSV: Left ventricular stroke volume
MCD: Mean contour distance;
MCD: Mean contour distanceNPV: Negative predictive value PPV: Positive predictive value
ReLU: Rectified linear unit
RV: Right ventricle/right ventricular
RVEDV: Right ventricular end-diastolic volume
RVEF: Right ventricular ejection fraction
RVESV: Right ventricular end-systolic volume
RVSV: Right ventricular stroke volume
SAD: Synthetically augmented dataset
SGD: Stochastic gradient descent
SV: Stroke volume
TGA: Transposition of the great arteries
TN: True negative
TOF: Tetralogy of Fallo
TP: True positive
